# PhaseTransfer: A transfer learning framework for efficient phase diagram mapping

**DOI:** 10.1038/s41524-026-02154-2

**Published:** 2026-05-26

**Authors:** Eduardo González-García, Albert J. Markvoort, Nadia A. Erkamp, Tom F. A. de Greef

**Affiliations:** 1 Synthetic Biology Group, Department of Biomedical Engineering, Eindhoven University of Technology, Eindhoven, The Netherlands; 2 Institute for Complex Molecular Systems, Department of Biomedical Engineering, Eindhoven University of Technology, Eindhoven, The Netherlands; 3 Laboratory of Chemical Biology, Department of Biomedical Engineering, Eindhoven University of Technology, Eindhoven, The Netherlands; 4 Institute for Molecules and Materials, Radboud University, Nijmegen, The Netherlands; 5 Center for Living Technologies, Eindhoven-Wageningen-Utrecht Alliance, Utrecht, The Netherlands

**Keywords:** Engineering, Materials science, Mathematics and computing

## Abstract

Self-driving laboratories accelerate materials discovery by autonomously designing and executing experiments through closed-loop integration of robotics and artificial intelligence. Active learning with Gaussian processes has enabled efficient phase diagram mapping, reducing required measurements by approximately 80% compared to conventional grid sampling. However, current approaches treat each system independently, discarding accumulated knowledge despite systematic similarities across related materials families. Here we introduce PhaseTransfer, a transfer learning framework that leverages previously characterized phase diagrams to accelerate mapping of new systems. PhaseTransfer combines a target model trained on current data with source models from related diagrams using spatially varying reliability assessments and focusing sampling on regions where transferred knowledge proves unreliable. Validation across synthetic benchmarks and experimental implementation on an autonomous microfluidic platform for biological condensate screening demonstrates an order of 50% reduction in sampling requirements compared to conventional active learning. By enabling knowledge reuse across investigations, transfer learning substantially enhances both the efficiency and generality of autonomous materials discovery.

## Introduction

Materials discovery has traditionally relied on laborious trial-and-error experimentation, often requiring decades to identify compounds with desired properties. This slow pace stems from the vastness of chemical and process parameter spaces and the high cost of systematic exploration. Self-driving laboratories (SDLs)—autonomous platforms that integrate robotic experimentation, real-time characterization, and artificial intelligence—are redefining this process by enabling closed-loop, data-driven discovery^1–3^. By autonomously designing experiments, executing them, and learning from the results in iterative cycles, SDLs explore chemical landscapes at speeds orders of magnitude faster than traditional workflows^4,5^.

In recent years, SDLs have evolved from proof-of-concept demonstrations into versatile discovery engines spanning a wide range of areas in materials science. Autonomous synthesis platforms have accelerated breakthroughs in inorganic materials^6,7^ and organic photovoltaics^8,9^. In energy research, robotic experimentation combined with intelligent optimization has enabled rapid tuning of electrolyte formulations and fast-charging protocols^10^. SDLs have also expanded into soft matter and biological systems, supporting curiosity-driven exploration of protocell formulations^11^, reinforcement-learning-guided flow chemistry^12^, and autonomous protein engineering^13^. In biophysics, microfluidic platforms integrated with adaptive algorithms now map phase separation in biomolecular condensates^14^, showcasing SDLs’ potential for studying intrinsically disordered protein systems. These platforms typically employ machine learning to balance exploration (searching novel, informative regions of parameter space) with exploitation (refining known promising areas), enabling efficient navigation of high-dimensional experimental landscapes^4,15–17^.

Among these applications, phase diagram construction—the systematic mapping of equilibrium and metastable phases as a function of composition, temperature, and processing conditions—represents a particularly important challenge. Automated phase diagram mapping is essential for understanding structure–property relationships and optimizing synthesis pathways. SDLs have demonstrated autonomous phase-diagram construction across diverse materials systems: Ament et al.^18^ introduced a platform that autonomously explores synthesis and annealing conditions to identify metastable inorganic phases, while the AMASE system^19^ integrates real-time experimentation with Bayesian inference to trace composition-temperature phase boundaries in metallic alloys. These advances confirm that SDLs can perform closed-loop, data-driven phase mapping. Yet, the experimental burden of characterizing phase boundaries remains substantial, motivating the development of more efficient sampling strategies.

Machine learning methods have substantially reduced sampling requirements for mapping complex phase diagrams. Active learning approaches have proven particularly effective. Dai and Glotzer^20^ demonstrated that Gaussian process (GP) models with uncertainty-based acquisition functions can prioritize the most informative experimental conditions—typically those near phase boundaries—enabling accurate phase diagram reconstruction using only ~20% of the measurements required by conventional grid sampling. Terayama et al. developed the Phase Diagram Construction (PDC) algorithm^21,22^, which combines label spreading with uncertainty sampling to improve interpolation across unmeasured regions. More recently, Zhu et al.^23^ introduced an expected-diagram-change acquisition strategy that explicitly targets experiments predicted to alter the interpolated phase diagram most significantly.

Despite these advances, current methods typically treat each phase diagram in isolation, discarding valuable knowledge from prior investigations. This approach overlooks a critical opportunity: phase diagrams within a given material family often share conserved topological features, similar boundary geometries, and systematic thermodynamic trends. These shared characteristics could accelerate the mapping of new systems through transfer learning (TL). In adjacent domains, TL has successfully exploited correlations across related systems, improving convergence rates and alleviating data scarcity in crystalline materials modeling^24^ and molecular property prediction^25–27^. Extending TL to phase-diagram mapping could dramatically enhance SDL efficiency by allowing platforms to build upon accumulated knowledge rather than learning each system from scratch.

**Fig. 1:**
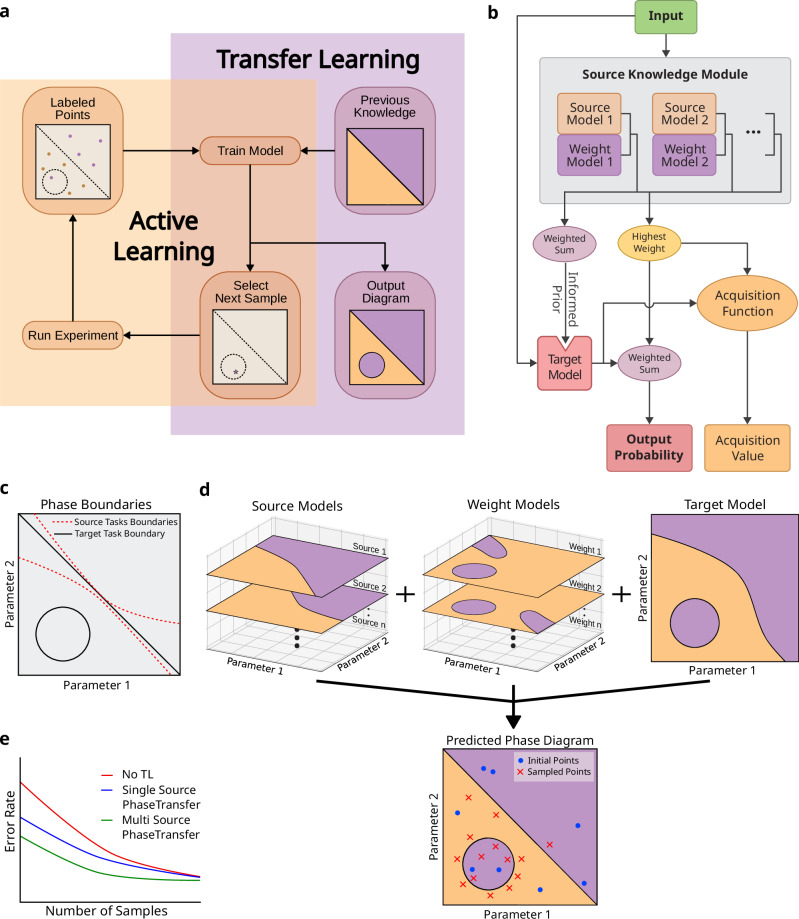
Multi-Source PhaseTransfer for efficient phase diagram mapping. **a** Integration of active learning with transfer learning. Left: Standard active learning loop—train model, select samples, run experiments, iterate. Right: Transfer learning enhancement—PhaseTransfer incorporates prior knowledge from related source systems, influencing both sampling strategy and predictions. **b** Multi-Source PhaseTransfer architecture. Input parameters flow through parallel pathways: a target model and a source knowledge module containing n source-weight model pairs. Each source model generates phase predictions, then its paired weight model computes a reliability score (0–1). The weighted combination of source predictions creates an informed prior for the target model, while the highest-weighted source prediction integrates with the target prediction to generate final phase classification and acquisition value for active learning optimization. **c** Illustrative target boundary (solid) with source boundaries (dashed) that partially align with different target regions. **d** Intended prediction and sampling behavior. Top panels show how components contribute: source models (left) provide prior phase predictions, weight models (center) identify regions where sources disagree with the target—notably at the novel circular boundary and misaligned corners—and the target model (right) refines predictions where sources prove unreliable. These combine into the final prediction (bottom). Blue dots: initial samples, red crosses: adaptively selected samples. Sampling concentrates near the novel circular boundary, while the diagonal—inferable from sources—requires fewer samples, enabling efficient exploration-exploitation balance. **e** Error convergence. Multi-Source PhaseTransfer (green) achieves the lowest initial error through transferred knowledge, Single-Source (blue) shows intermediate performance, vanilla active learning (red) starts with the highest. All converge to a comparable final error rate, but transfer learning methods reach low error thresholds faster.

Here, we introduce PhaseTransfer, a transfer learning framework (Fig. 1) that extends GP active learning by leveraging previously characterized phase diagrams. PhaseTransfer combines a model of the current system with source models from related diagrams, using spatially varying weight models to assess where prior knowledge is reliable and concentrating sampling on regions where it proves uninformative (Fig. 1c, d). This exploration-exploitation balance accelerates convergence compared to learning from scratch (Fig. 1e). We also present an automated source-pruning algorithm that filters redundant sources, preserving ensemble diversity.

We validate PhaseTransfer across three families of synthetic systems and experimentally using PhaseXplorer^14^, an autonomous microfluidic platform for mapping biomolecular condensate phase separation. Across all benchmarks, PhaseTransfer reduces sampling requirements on the order of 50% compared to conventional active learning while maintaining or improving accuracy.

## Results

### PhaseTransfer overview

PhaseTransfer extends GP active learning for binary phase classification^20^ by incorporating prior knowledge from previously characterized phase diagrams (Fig. 1a). The framework combines three components: a target model trained on observations from the system under investigation, one or more source models derived from related phase diagrams, and weight models that evaluate where each source reliably predicts the target system’s phases.

Source-weight model pairs serve two functions (Fig. 1b). First, they construct a spatially informed prior for the target model: where a source is deemed reliable, its posterior mean seeds the target GP prior, accelerating convergence; where it is unreliable, the prior reverts to the uninformed baseline. Second, they contribute to the final phase prediction through a weighted combination with the target model, using an adaptive exponent that links source confidence to a required reliability level, preventing overconfident source models from skewing phase predictions. In the multi-source setting, different sources may capture complementary portions of the target parameter space (Fig. 1c). At each location, the weight models select the most reliable source for prediction while jointly contributing to the informed prior (Fig. 1d).

The resulting acquisition function couples predictive uncertainty with spatially resolved source reliability. Where source trust exceeds a threshold, sampling shifts toward unexplored territory rather than redundantly characterizing boundaries already captured by source knowledge; where trust is low, the standard uncertainty-driven strategy applies. Together, these mechanisms accelerate convergence compared to learning from scratch (Fig. 1e). To handle large source libraries, an automated pruning algorithm filters diagrams with significant topological and geometric overlap, retaining a diverse subset that captures distinct phase behaviors without redundancy. The complete mathematical formulation is provided in “Methods”.

We validate PhaseTransfer across two complementary settings. First, we evaluate performance on three families of synthetic phase diagrams: explicitly defined phase boundaries, simplified three-component biological condensate simulations, and mass-balance models of supramolecular copolymerization. For all three families, ground truth is fully known, enabling precise quantitative comparison against baseline methods across 50 independent runs. Second, we validate the framework experimentally by integrating PhaseTransfer within PhaseXplorer^14^, an autonomous microfluidic platform for mapping biomolecular condensate phase separation, demonstrating robustness to real-world measurement noise and experimental variability. For the synthetic benchmarks, we compare Single- and Multi-Source PhaseTransfer against three state-of-the-art methods: vanilla GP active learning^20^, GP with expected change acquisition function (GP-ECA)^23^, and the PDC algorithm^21^, quantifying performance using the error rate: the fraction of grid points where the predicted phase label disagrees with ground truth. The evaluation protocol is detailed in “Methods”.

### Synthetic benchmark: sine wave phase diagram

**Fig. 2:**
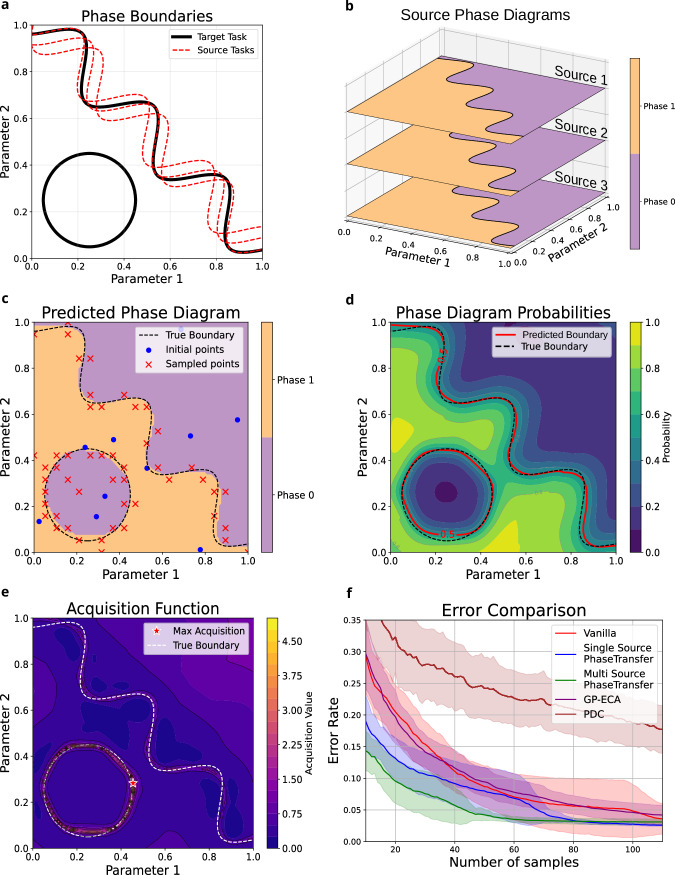
Sine wave phase diagram benchmark. **a** Schematic of phase boundaries of target (solid) and three sources (dashed) with different phase shifts. Each source aligns with different regions of the target, and the circular boundary is absent from all sources. **b** The three source phase diagrams (purple: Phase 0, orange: Phase 1). **c** Multi-Source PhaseTransfer phase diagram reconstruction after 50 samples (black dashed line: true boundary, blue dots: 10 initial random samples, red crosses: 50 actively selected samples). Despite the sinusoidal boundary’s greater complexity, sampling density is comparable between both boundaries, as the sinusoidal boundary is inferable from sources. **d** Multi-Source PhaseTransfer phase probability prediction at iteration 50. The predicted boundary (*p* = 0.5) closely matches the ground truth. **e** Multi-Source PhaseTransfer acquisition function at iteration 50. High values concentrate on the novel circular boundary, while low values along the sinusoid reflect confidence in transferred knowledge. The starred maximum marks the next sampling location—on the circular boundary. **f** Error rate comparison across five active learning methods (averages of 50 independent runs, shading: ±1 standard deviation). TL methods exhibit lower initial error rate and converge to comparable final accuracy. Multi-Source PhaseTransfer (green) consistently outperforms baseline methods throughout the active learning process. The circular boundary—absent from all sources—is reliably identified, demonstrating robustness to partial source-target mismatch.

As a first test of PhaseTransfer, we consider phase diagrams with sinusoidal boundaries. We construct three source diagrams using sine functions with different phase shifts (Fig. 2a). The target diagram features a sine boundary with an increased period that partially aligns with different regions of each source. Additionally, a circular phase boundary absent from all source tests tests the method’s ability to identify novel features. We generate source models by applying standard active learning to the source diagrams (Fig. 2b), then evaluate PhaseTransfer against baseline methods under identical sampling conditions across 50 independent runs.

To illustrate the method’s behavior, we examine a representative Multi-Source PhaseTransfer run after 50 iterations starting from 10 random points. The predicted phase diagram accurately reconstructs both boundaries, matching ground truth closely (Fig. 2c). Despite the sinusoidal boundary’s greater length and complexity, both boundaries receive comparable sample numbers because regions inferable from source knowledge require fewer measurements, while the novel circular feature demands direct exploration. The probability map (Fig. 2d) shows close alignment between the predicted decision boundary (*p* = 0.5) and true phase boundaries. The acquisition function (Fig. 2e) quantifies where sampling would be most informative: high acquisition values concentrate around the novel circular boundary, while notably lower values appear near the sinusoidal boundary, reflecting confidence in transferred source knowledge.

Aggregating results across all 50 runs reveals that Multi-Source PhaseTransfer consistently outperforms baseline approaches (Fig. 2f, Table 1). The advantage emerges immediately: at the first iteration, Multi-Source PhaseTransfer exhibits substantially lower error (~15%) than vanilla active learning (~30%), demonstrating that transferred knowledge provides immediate benefits before any active sampling. Multi-Source PhaseTransfer achieves the fastest convergence, requiring 57% fewer samples than vanilla active learning to reach 10% error and 56% fewer to reach 5% error. Single-Source PhaseTransfer, using only Source 1 (Fig. 2b, top), shows intermediate performance (35 samples to 10% error), demonstrating that incorporating multiple related source diagrams provides additional benefit beyond a single source.

**Table 1:** Performance comparison of active learning methods on the sine wave phase diagram benchmark

	Avg number of samples before		
Model	<0.1 Error	<0.05 Error	Final error
Vanilla	44 (>110)	99 (>110)	0.036
Single-Source PhaseTransfer	35 (71)	68 (78)	**0.026**
Multi-Source PhaseTransfer	**19 (47)**	**44 (58)**	0.031
GP-ECA	47 (>110)	92 (>110)	0.042
PDC	>110 (>110)	>110 (>110)	0.178

Error rates represent the fraction of misclassified points on a 100 × 100 test grid, averaged over 50 independent runs. Bold values indicate the best performance. Values inside parentheses indicate the worst runs.

Multi-Source PhaseTransfer maintains superior performance even in worst-case scenarios. In the worst runs (parenthetical values in Table 1), vanilla and GP-ECA methods fail to identify the circular boundary within the 110 samples in 5% of runs, while TL methods consistently achieve complete characterization within this limit. GP-ECA provides only marginal improvement over vanilla despite its sophisticated acquisition function. PDC substantially underperforms all other methods, failing to reach 10% error after 110 samples and achieving 18.5% final error–suggesting fundamental limitations in its applicability to binary phase diagram mapping compared to GP-based methods.

The Supplementary Information provides extended performance evaluations on this benchmark. Uncertainty-guided acquisition outperforms classical space-filling sampling strategies, such as random, Latin hypercube^28^, and minimax^29^ sampling; this advantage persists even when space-filling is combined with source knowledge, confirming the benefit of model-informed over geometry-based approaches (SI Section 4.1, Figure S2). PhaseTransfer also outperforms Multi-Task Gaussian processes^30^, an alternative transfer learning approach used in regression-based Bayesian optimization^31^, which fails to effectively exploit source knowledge for binary phase diagram mapping (SI Section 4.2, Figure S3). Under noisy observations, PhaseTransfer consistently maintains its efficiency advantage over baseline approaches across all tested noise combinations (SI Section 4.3, Figure S4), with input shift contributing more to performance degradation than phase misclassification noise. Finally, systematic analysis of source-target boundary misalignment shows that Single-Source PhaseTransfer efficiency depends jointly on alignment degree and the geometric complexity of unreliable regions: partially misaligned sources still outperform vanilla active learning once unreliable regions are resolved, while complete misalignment returns performance equivalent to learning from scratch (SI Section 4.4, Figure S5).

### Synthetic benchmark: three-component biological condensate

The second family of phase diagrams involves biological condensates–membraneless compartments that form through liquid–liquid phase separation of proteins and nucleic acids, playing crucial roles in cellular organization and function^32,33^. Understanding their thermodynamic landscape has become increasingly important, with recent studies revealing that condensate formation depends sensitively on multicomponent composition^34^ and that complex energy landscapes govern nucleation pathways^35^. A key feature of this landscape is the spinodal region, a thermodynamically unstable zone where spontaneous phase separation occurs without nucleation^36^. Mapping these phase boundaries is essential for understanding condensate assembly mechanisms and developing therapeutic strategies targeting condensate-related diseases^37,38^. Yet, complete experimental characterization remains challenging: systematic exploration of multi-dimensional parameter spaces (protein concentration, RNA concentration, ionic strength, pH, crowding agents) requires hundreds to thousands of individual measurements^39^, though recent active learning approaches show promise^40^. This substantial experimental burden, combined with the biological and therapeutic importance of accurate phase boundary determination, makes biological condensates an ideal test case for our TL approach.

**Fig. 3:**
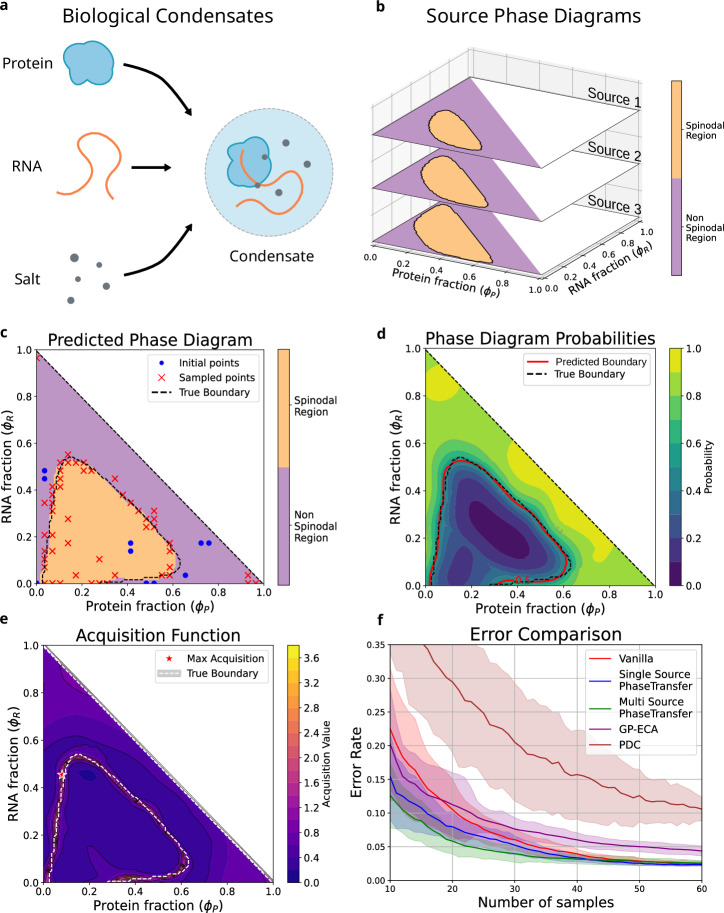
Three-component biological condensate benchmark. **a** Condensate system (protein, RNA, salt) modeled via Flory–Huggins theory. **b** Source phase diagrams at ionic strengths *I* = 0.3 (bottom), 0.45 (middle), and 0.6 M (top), showing evolution of the spinodal boundary. Orange: spinodal (phase-separated), purple: non-spinodal (mixed). All exhibit reentrant behavior where intermediate protein and RNA concentrations favor phase separation. **c** Multi-Source PhaseTransfer prediction for target system (*I* = 0.75 M) after 50 samples. Axes: protein (*ϕ*_*P*_) and RNA (*ϕ*_*R*_) fractional concentrations, black dashed line: true boundary, blue dots: 10 initial random samples, red crosses: 50 actively selected samples. The reentrant boundary shape is captured with focused sampling along the phase boundary. **d** Multi-Source PhaseTransfer probability map at iteration 50. The predicted boundary (*p* = 0.5) aligns well with the ground truth. **e** Multi-Source PhaseTransfer acquisition function, where high values identify optimal sampling locations. The starred maximum marks the next sampling location. **f** Error rate comparison across five active learning methods (averages of 50 independent runs, shading: ±1 standard deviation, cropped 140 × 140 triangular grid respecting composition constraints). TL methods exhibit a lower initial error rate and converge to comparable final accuracy. Multi-Source PhaseTransfer (green) consistently outperforms baseline methods throughout the active learning process.

This benchmark employs Flory–Huggins theory^41,42^ to model the spinodal region of a simplified three-component biological condensate comprising protein, RNA, and salt, with fractional concentrations *ϕ*_*P*_, *ϕ*_*R*_, and *ϕ*_*S*_ (Fig. 3a). The system’s free energy follows:

1 $$\begin{array}{l}\frac{F}{{k}_{B}T}=\frac{{\phi }_{P}}{{N}_{P}}\,{\rm{ln}}{\phi }_{P}+\frac{{\phi }_{R}}{{N}_{R}}{\rm{ln}}{\phi }_{R}+\frac{{\phi }_{S}}{{N}_{S}}{\rm{ln}}\,{\phi }_{S}\\ +{\chi }_{PR}(I){\phi }_{P}{\phi }_{R}+{\chi }_{PS}(I){\phi }_{P}{\phi }_{S}+{\chi }_{RS}(I){\phi }_{R}{\phi }_{S}\end{array}$$

where *k*_*B*_ is Boltzmann’s constant, *T* is temperature, and *N*_*P*_ = 200, *N*_*R*_ = 100, and *N*_*S*_ = 1 represent monomer chain lengths. The parameters *χ*_*PR*_, *χ*_*PS*_, and *χ*_*RS*_ (SI Section 2) describe pairwise interaction strengths between components, depending on ionic strength *I*; varying *I* generates different energy landscapes. Given fractional concentrations *ϕ*_*P*_ and *ϕ*_*R*_, we calculate *ϕ*_*S*_ = 1 − *ϕ*_*P*_ − *ϕ*_*R*_ and classify this 2D point as inside or outside the spinodal region by examining the free energy Hessian: the point lies outside if the Hessian is positive semidefinite, inside otherwise. We generate three source phase diagrams at ionic strengths *I* ∈ {0.3, 0.45, 0.6} (Fig. 3b) and construct the target diagram at *I* = 0.75. We again evaluate PhaseTransfer against baseline active learning methods under identical sampling conditions across 50 independent runs.

A representative Multi-Source PhaseTransfer run after 50 iterations successfully captures the characteristic reentrant spinodal boundary shape, with focused sampling along the boundary and close alignment between predicted and true boundaries (Fig. 3c–e). Aggregating results across all 50 runs reveals that Multi-Source PhaseTransfer consistently outperforms baseline approaches (Fig. 3f, Table 2). Multi-Source PhaseTransfer exhibits lower initial error and achieves faster convergence, requiring 38% fewer samples than vanilla active learning to reach 10% error and 32% fewer to reach 5% error. Single-Source PhaseTransfer (using Source 3 at *I* = 0.6 M) shows intermediate performance, demonstrating that incorporating multiple source diagrams provides additional benefit. GP-ECA shows minimal improvement over vanilla, while PDC substantially underperforms, failing to reach 10% error within 60 samples.

**Table 2:** Performance comparison of active learning methods on the three-component biological condensate benchmark

	Avg Number of Samples Before		
Model	<0.1 Error	<0.05 Error	Final error
Vanilla	21 (31)	34 (43)	0.025
Single-Source PhaseTransfer	16 **(26)**	31 (42)	**0.023**
Multi-Source PhaseTransfer	**13** (32)	**23 (34)**	0.026
GP-ECA	23 (34)	51 (>60)	0.044
PDC	>60 (>60)	>60 (>60)	0.106

Error rates represent the fraction of misclassified points on a 100 × 100 test grid, averaged over 50 independent runs. Bold values indicate the best performance. Values inside parentheses indicate the worst runs.

### Synthetic benchmark: supramolecular copolymerization

The third family of phase diagrams involves supramolecular polymers–long, one-dimensional assemblies formed through reversible non-covalent interactions^43,44^. These systems exhibit unique properties including self-healing, stimuli-responsiveness, and recyclability, making them attractive for applications from biomedicine to functional materials^45^. Unlike conventional polymers, supramolecular systems undergo dynamic monomer exchange, with highly cooperative assembly behavior governed by thermodynamic equilibria sensitive to temperature, concentration, and solvent composition, often producing sharp polymerization transitions^46^. For copolymerizations, characterization is further complicated by the delicate balance between competing homo- and hetero-interactions^47,48^. Mapping how system parameters (monomer concentrations, temperature, equilibrium constants) influence polymerization degree and aggregate distributions requires extensive titration and cooling experiments^49^. This experimental burden makes supramolecular polymerization another valuable test case, demonstrating transfer learning across diverse scientific domains.

This benchmark employs the mass-balance model of supramolecular copolymerization by ten Eikelder et al.^50^, which describes the system as equilibrium reactions between monomers and all possible aggregates. Mass-balance constraints require each monomer to exist either free or within an aggregate. Because equilibrium concentrations of all aggregates can be expressed in terms of free monomer concentrations via equilibrium constants, the model yields *n* equations for *n* free monomer concentrations. Solving this system numerically determines the complete equilibrium composition, including the total polymer concentration *P*_tot_. For details on the mass-balance equations, see SI Section 3, Eqs. (4)–(11).

**Fig. 4:**
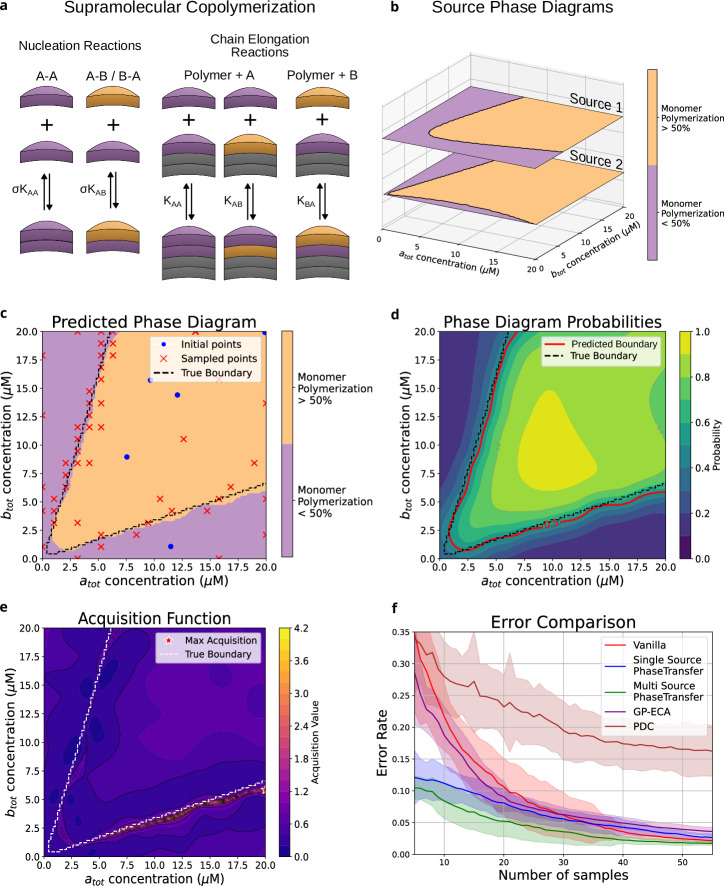
Supramolecular copolymerization benchmark. **a** Reaction scheme of two-component supramolecular copolymerization with forbidden B–B bonds. Purple and orange shapes represent monomers A and B, respectively. Nucleation (dimer formation) is a factor *σ* weaker than chain elongation. The equilibrium constants depend on the monomer types involved in the newly formed bond. **b** Source phase diagrams with varying equilibrium constants, defined relative to *K*_*e*_ = 10^6^ M^−1^, i.e., *K*_*AA*_ = *f*_*AA*_*K*_*e*_, *K*_*AB*_ = *K*_*BA*_ = *f*_*AB*_*K*_*e*_. Top: *f*_*AA*_ = 0.01, *f*_*AB*_ = 0.5 (weak homopolymerization, moderate copolymerization). Bottom: *f*_*AA*_ = 0.1, *f*_*AB*_ = 5 (moderate homopolymerization, strong copolymerization). Orange: polymer-dominated, purple: monomer-dominated. Axes: initial concentrations (0–20 μM). **c** Multi-Source PhaseTransfer phase diagram prediction for target system governed by weak A homopolymerization and strong copolymerization (*f*_*AA*_ = 0.01, *f*_*AB*_ = 5) after 50 samples. Black dashed line: true boundary, blue dots: 5 initial, red crosses: 50 actively selected samples. **d** Multi-Source PhaseTransfer probability map at iteration 50. The predicted boundary (*p* = 0.5) accurately reproduces the ground truth. **e** Multi-Source PhaseTransfer acquisition function, where high values identify optimal sampling regions. The starred maximum marks the next sampling location. **f** Error rate comparison across five active learning methods (averages of 50 independent runs, shading: ±1 standard deviation). Strong source-target correlation enables Multi-Source PhaseTransfer (green) to achieve significantly lower initial error rate compared to vanilla baseline. All GP-based methods converge rapidly after 30 samples.

We consider a two-monomer system (A and B) following a nucleation-elongation (cooperative) polymerization mechanism, where monomers form dimers (nucleation) or elongate polymer chains (Fig. 4a). The cooperativity factor *σ* distinguishes this from isodesmic polymerization: dimer formation is governed by weaker equilibrium constants *σK*_*AA*_ (A–A binding), *σK*_*AB*_ (A–B binding), *σK*_*BA*_ (B–A binding), and *σK*_*BB*_ (B–B binding), while chain elongation proceeds via stronger constants *K*_*AA*_, *K*_*BA*_, *K*_*AB*_, and *K*_*BB*_. This nucleation barrier produces sharper phase boundaries than isodesmic systems. We constrain the system so that B–B bonds are forbidden (*K*_*BB*_ = 0) and A–B binding is symmetric (*K*_*BA*_ = *K*_*AB*_), then we define a base equilibrium constant *K*_*e*_ and two fractional factors *f*_*AA*_ and *f*_*AB*_ to parameterize the system:

2 $$\left\{\begin{array}{ll}{K}_{e} & ={10}^{6}\,{M}^{-1},\sigma ={10}^{-5}\\ {K}_{AA} & ={f}_{AA}{K}_{e}\\ {K}_{AB} & ={K}_{BA}={f}_{AB}{K}_{e}\\ {K}_{BB} & =0\end{array}\right.$$

Given initial monomer concentrations *a*_tot_ and *b*_tot_, the model computes equilibrium polymer concentrations, returning total polymer concentration *P*_tot_. We define a binary phase based on whether more than half the monomers have polymerized. We use two source systems: one with very low A homopolymerization and moderate AB copolymerization (*f*_*AA*_ = 0.01, *f*_*AB*_ = 0.5), another with higher A homopolymerization and strong AB copolymerization (*f*_*AA*_ = 0.1, *f*_*AB*_ = 5) (Fig. 4b). The target system features very low A homopolymerization with strong AB copolymerization (*f*_*AA*_ = 0.01, *f*_*AB*_ = 5). We again evaluate PhaseTransfer against baseline active learning methods under identical sampling conditions across 50 independent runs.

A representative Multi-Source PhaseTransfer run after 50 iterations accurately captures the target phase boundary, with close alignment between predicted and true values (Fig. 4c–e). Aggregating results across all 50 runs reveals that Multi-Source PhaseTransfer consistently outperforms baseline approaches (Fig. 4f, Table 3). Strong correlation between source and target systems enables Multi-Source PhaseTransfer to exhibit substantially lower initial error and achieve faster convergence, requiring 67% fewer samples than vanilla active learning to reach 10% error and 37% fewer to reach 5% error. Single-Source PhaseTransfer (using Source 1 with *f*_*AA*_ = 0.01, *f*_*AB*_ = 0.5) shows intermediate performance, demonstrating that incorporating both source diagrams provides additional benefit. GP-ECA shows moderate improvement over vanilla, while PDC substantially underperforms, failing to reach 10% error within 55 samples.

**Table 3:** Performance comparison of active learning methods on the supramolecular copolymerization benchmark

	Avg number of samples before		
Model	<0.1 Error	<0.05 Error	Final error
Vanilla	21 (39)	35 (42)	0.021
Single-Source PhaseTransfer	14 (28)	34 (49)	0.026
Multi-Source PhaseTransfer	**7** (39)	**22 (40)**	**0.017**
GP-ECA	19 **(27)**	40 (56)	0.036
PDC	>55 (>55)	>55 (>55)	0.162

Error rates represent the fraction of misclassified points on a 100 × 100 test grid, averaged over 50 independent runs. Bold values indicate the best performance. Values inside parentheses indicate the worst runs.

To explore robustness to irrelevant source knowledge, we evaluate Multi-Source PhaseTransfer on this benchmark with varying numbers of irrelevant source diagrams (SI Section 4.5, Fig. S6). When at least two relevant sources are present, adding several irrelevant sources has no considerable impact on the final mapping efficiency, with error curves clustering entirely by the number of relevant sources. Performance approaches or falls below the vanilla baseline only in extreme scenarios where irrelevant sources vastly outnumber relevant ones and simultaneously output highly confident incorrect predictions; incorporating multiple relevant sources provides the most effective mitigation in this scenario. These results demonstrate that weight models successfully identify and suppress unreliable sources without manual curation.

### Autonomous phase mapping of biomolecular condensate

Having established transfer learning effectiveness on synthetic benchmarks, we now validate it experimentally. We integrated PhaseTransfer within PhaseXplorer^14^, an autonomous microfluidic platform for mapping biomolecular condensate phase separation, to obtain binary phase diagrams of BSA (bovine serum albumin) and PEG (polyethylene glycol). At sufficiently high concentrations (threshold depending on pH, PEG molecular weight, and salt concentration), these components undergo segregative phase separation, forming two liquid-like phases: one enriched in PEG, one enriched in BSA^51,52^. The BSA-enriched phase is termed the condensate.

**Fig. 5:**
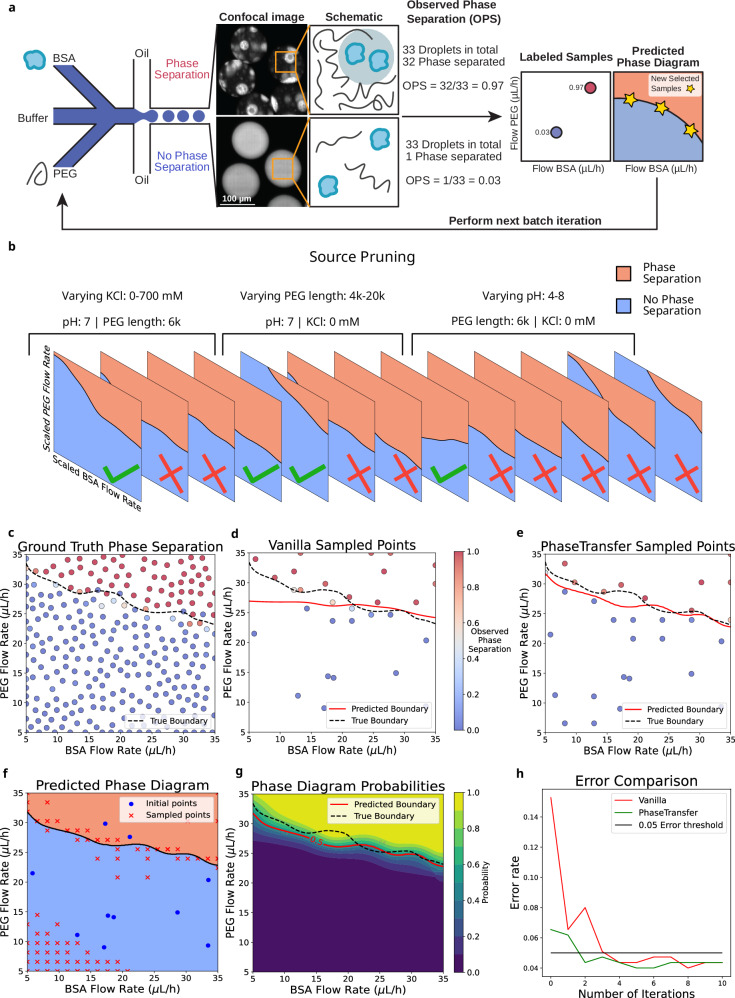
Experimental validation with PhaseXplorer. **a** PhaseXplorer workflow. This autonomous platform uses microfluidic technology to prepare droplets that can phase separate (top) or stay homogeneous (bottom) (SI Section 5, Fig. S8). The platform generates 30–100 droplets per experimental condition and determines the observed phase separation fraction (OPS). Then, either vanilla active learning or PhaseTransfer predicts the full phase diagram and selects the next batch of experimental conditions without human intervention. **b** Source library pruning. From 13 BSA-PEG phase diagrams spanning salt concentration (0–700 mM KCl), PEG length (4–20 k), and pH (4–8), the pruning algorithm selected 4 diverse sources: PEG 6k/pH 7/0 mM, PEG 6k/pH 7/700 mM, PEG 4k/pH 7/0 mM, and PEG 20k/pH 7/0 mM KCl. **c** Ground truth for target system (PEG 8k/pH 4.7/0 mM KCl) from 453 autonomous measurements. Color intensity represents OPS from PhaseXplorer (blue: homogeneous, red: phase-separated). The phase boundary (dashed line) occurs at PEG flow rates of 22–35 μL/h depending on BSA flow rate, exhibiting the characteristic nonlinear shape of protein-polymer systems. **d**, **e** Comparison after 2 batch iterations (30 samples: 10 initial and 10 per batch). Vanilla active learning (**d**) shows predicted boundary (solid red) misalignment with the true boundary (dashed) at low BSA flow rates due to a lack of information. PhaseTransfer (**e**) overcomes this trade-off and predicts the full boundary using transferred knowledge. **f** Final PhaseTransfer prediction after 10 iterations (110 samples). Blue dots: 10 initial samples, red crosses: 100 actively selected samples. Predicted boundary closely matches ground truth. **g** Final PhaseTransfer probability map. High confidence (*p* near 0 or 1) except along the true phase boundary (dashed line), where a thin uncertainty band reflects inherent experimental variability in measuring OPS. **h** Error rate comparison, calculated as the misclassification rate of measured ground truth samples. PhaseTransfer (green) reaches 5% error in 2 batch iterations versus 4 for vanilla—a twofold reduction in experimental burden.

PhaseXplorer employs microfluidic technology to flow aqueous solutions containing BSA, buffer, and PEG into oil, encapsulating them into droplets (Fig. 5a). The flow rates of BSA and PEG solutions (5–35 μL/h for each component) directly determine droplet composition, defining a 2D parameter space analogous to our synthetic benchmarks. After mixing and incubation, droplets are imaged with a confocal fluorescence microscope. A fluorescent dye conjugated to BSA enables direct visualization of phase separation: homogeneous droplets show uniform fluorescence, while phase-separated droplets exhibit distinct bright and dim regions corresponding to condensate and dilute phases, respectively. PhaseXplorer creates many droplets for each composition and automatically analyzes their images, calculating the observed phase separation fraction (OPS)–defined as the ratio of phase-separated droplets to total droplets for each experimental composition. Representative confocal images illustrate the clear visual distinction between these states across the full composition space (SI Section 5, Fig. S8). Conditions yielding OPS > 0.5 are classified as phase-separated, producing binary phase diagrams. This autonomous characterization enables closed-loop active learning: an algorithm selects the next experimental conditions, PhaseXplorer executes experiments, and OPS measurements update the model.

We compare two active learning strategies: vanilla GP^20^ and Multi-Source PhaseTransfer (hereafter simply PhaseTransfer). Due to experimental constraints, we focus on these two methods rather than the full comparison performed on synthetic benchmarks. For both methods, each iteration selects a batch of 10 samples by identifying points that maximize acquisition value while maintaining a minimum separation distance *r* = 0.1 (corresponding to 4.5 μL/h). Batch sampling improves experimental throughput while yielding comparable performance to single-point iteration (SI Section 4.6, Fig. S7). We use a library of 13 BSA-PEG phase diagrams as source knowledge, spanning salt concentration (0–700 mM KCl), PEG length (4–20k), and pH (4–8). Our source pruning algorithm (see Methods) reduces this library to four diverse representatives: PEG 6k/pH 7/0 mM KCl, PEG 6k/pH 7/700 mM KCl, PEG 4k/pH 7/0 mM KCl, and PEG 20k/pH 7/0 mM KCl (Fig. 5b). The target system is BSA-PEG at PEG 8k/pH 4.7/0 mM KCl, which has not previously been measured. Ground truth was established through exhaustive autonomous sampling of 453 compositions (Fig. 5c and SI Fig. S8). Error rate is calculated as the fraction of ground truth compositions misclassified by the predicted phase diagram.

After two iterations, both methods (Fig. 5d, e) accurately predict the phase boundary at high BSA flow rates. However, vanilla active learning fails to infer the boundary at low BSA flow rates due to insufficient sampling. In contrast, PhaseTransfer correctly predicts this region by leveraging transferred knowledge from related source diagrams with phase boundaries in similar locations. Final PhaseTransfer predictions after 10 iterations show close alignment between predicted and ground truth boundaries, with minor deviations attributable to inherent experimental noise (Fig. 5f, g). Samples accumulate in the bottom-left corner during later iterations (Fig. 5f) because the batch sampling protocol continues selecting 10 points per iteration even after complete boundary characterization, directing samples to regions with marginally higher but ultimately uninformative acquisition values.

Error rates across iterations show that PhaseTransfer reaches the target error threshold of 0.05 within 2 batch iterations, while vanilla active learning requires 4 iterations (Fig. 5h). This two-fold acceleration demonstrates practical advantages for expensive experimental systems. Beyond iteration 4, both methods converge to comparable accuracy, having successfully characterized this system’s relatively simple phase boundary structure.

## Discussion

Despite decades of accumulated phase diagram data across chemistry, materials science, and biophysics, each new system is typically characterized from scratch, requiring hundreds of measurements even when similar systems have been extensively studied. We have introduced PhaseTransfer, a transfer learning framework that systematically utilizes existing knowledge to accelerate phase diagram mapping. Our validation strategy balances rigorous quantitative assessment through synthetic benchmarks, where ground truth enables precise evaluation, with experimental validation on an autonomous BSA-PEG phase-separation study using PhaseXplorer, demonstrating robustness to real-world measurement noise and experimental variability. Across all benchmarks, PhaseTransfer consistently outperforms baseline approaches through three mechanisms: informative priors that reduce initial error, weighted ensemble predictions that leverage both source and target model knowledge, and adaptive sampling strategies that accelerate convergence. Together, these achieve approximately 50% reduction in sampling requirements compared to conventional active learning.

The conceptual advance of PhaseTransfer lies in constructing data-driven priors from empirical observations of related systems, rather than encoding explicit physics models as in recent physics-informed GP methods for phase diagram construction^53,54^. This distinction proves advantageous when rigorous physics models are unavailable, underdeveloped, or computationally prohibitive, situations common in complex biological condensates, metastable materials, and supramolecular assemblies, where thermodynamic modeling faces fundamental challenges. Transfer learning proves effective in these settings because related systems within a material family share conserved topological features and boundary geometries arising from similar underlying physics. Source models capture these patterns while weight models assess where they reliably apply to new targets, enabling knowledge transfer even when sources and target systems differ in details. The sine wave benchmark demonstrates this tolerance: sources missing the novel circular boundary still accelerated mapping, with each source’s distinct phase shift informing different target regions and providing complementary knowledge.

Because PhaseTransfer’s effectiveness depends on the quality and diversity of the source ensemble, thoughtful source selection is central to its practical deployment. Domain knowledge identifying systems with shared physical mechanisms provides the most reliable guidance; when such knowledge is unavailable, the optimal strategy is to apply source pruning to filter redundant diagrams from a complete library, then incorporate all retained sources. This approach is supported by PhaseTransfer’s robust handling of negative transfer (SI Section 4.5, Fig. S6): spatially varying weight models identify and suppress unreliable sources, and adding several irrelevant sources has no considerable impact on the final mapping efficiency when at least two relevant sources are present. Performance approaches or falls below the vanilla baseline only in extreme scenarios where irrelevant sources vastly outnumber relevant ones and output highly confident, incorrect predictions. Such configurations are unlikely when following domain knowledge or pruning guidelines. Diminishing returns naturally emerge once the source ensemble jointly covers the target parameter space, beyond which additional sources yield no further gain. Since the optimal subset cannot be identified a priori, the recommended strategy is to incorporate all retained sources and allow the weight models to select locally. In practical deployment scenarios, where the source pool contains at least some related systems and unrelated sources do not exhibit extreme uncorrelation with the target, this approach consistently equals or exceeds learning from scratch.

Beyond source selection, PhaseTransfer is designed with model-agnostic flexibility to accommodate diverse experimental contexts. Source and weight models require only that they output phase probabilities, while target models must additionally provide uncertainty estimates for acquisition function evaluation. While we employ Gaussian processes throughout for their natural uncertainty quantification and suitability for sparse sampling regimes, practitioners can substitute domain-specific or computationally efficient alternatives as needed, making the framework adaptable to any data pipeline.

This work focuses on two-dimensional binary phase diagrams, and two natural extensions warrant investigation: higher-dimensional spaces and the prediction of more than two phases. Higher-dimensional parameter spaces present no fundamental algorithmic barriers, as the Gaussian process formulation, acquisition functions, and weight model training generalize naturally to arbitrary dimensions; the primary challenge remains the curse of dimensionality that affects all active learning methods, though transfer learning partially mitigates this through informative priors. Extending to systems with more than two phases can be approached in three ways. The first approach uses one-versus-rest decomposition: independent models distinguish each phase from all others, and points are assigned to the phase with the highest predicted probability. This preserves the full GP performance benefits, but incurs multiplicative computational cost (*k* target models, *k* × *n* source models, and *k* × *n* weight models for *k* phases and *n* sources). The second approach replaces GP surrogate models with natively multi-class classifiers, reducing computational cost but potentially sacrificing the performance advantages that GPs offer in sparse sampling regimes. The third, a hybrid approach, employs lightweight multi-class models for source and weight predictions while reserving GPs for the target model, offering a promising path toward efficient scaling that balances accuracy and computational cost. Diagrams with phase coexistence regions also fit naturally into all three strategies: either the coexistence region can be defined as an additional phase class, in which case all three approaches apply directly, or the problem can be framed such that multiple phases can be predicted simultaneously, supported by both one-versus-rest configurations and some natively multi-class models. Finally, while our experimental validation demonstrates real-world applicability, it covers a single system (BSA-PEG) with a relatively simple phase boundary; future work should test systems with more complex topologies and multiple disconnected phase regions.

Taken together, these results demonstrate that transfer learning can fundamentally enhance autonomous phase diagram mapping through systematic knowledge reuse. To support adoption, we provide phaseGP, a Python library built with GPyTorch^55^ that implements both standard GP active learning and Multi-Source PhaseTransfer, supporting scikit-learn-compatible classifiers^56^ as source models. As autonomous laboratories generate expanding collections of characterized phase diagrams, each completed investigation enriches the shared knowledge base, accelerating subsequent explorations. In multi-laboratory settings, this architecture could enable federated networks where institutions collectively refine phase boundaries by pooling diagrams collected under different conditions, amplifying knowledge transfer across research communities. This compounding effect positions self-driving laboratories not merely as automated experimentation platforms, but as adaptive, interconnected learning systems that grow more efficient with experience.

## Methods

### Overview

This section provides the complete mathematical formulation of the PhaseTransfer framework. We first establish the Gaussian process active learning foundation for binary phase classification, then detail the transfer learning equations for single-source and multi-source scenarios, describe the source pruning algorithm, and finally present the benchmark evaluation protocol.

### Active learning framework

We consider a parameter space $X\subseteq {{\mathbb{R}}}^{d}$ where each point *x* ∈ *X* represents experimental conditions such as temperature, pressure, or composition. An experiment or simulation at location *x* determines the phase label *y*(*x*) ∈ {0, 1}, where 0 and 1 denote the two distinct phases.

Gaussian processes (GPs) suit this task because they provide both predictions and uncertainty quantification. A GP defines a distribution over functions $f:X\to {\mathbb{R}}$, specified by a mean function *μ*(*x*) and covariance function $k(x,{x}^{{\prime} })$. After observing data ${\mathcal{D}}=\{({x}_{1},{y}_{1}),...,({x}_{t},{y}_{t})\}$, the GP posterior provides a predictive distribution for any new point x^*^, yielding a mean prediction $\mu ({x}^{* }| {\mathcal{D}})$ and predictive variance ${\sigma }^{2}({x}^{* }| {\mathcal{D}})$ that quantify model confidence.

For binary classification, we model the latent function *f*(*x*) through a GP and link it to binary observations via a probit transformation: *P*(*y* = 1∣*x*) = *Φ*(*f*(*x*)), where *Φ* denotes the standard normal cumulative distribution function (CDF). During training, we transform binary labels {0, 1} into a latent representation with negative and positive values, such as $\{\log \frac{\epsilon }{1+\epsilon },\log \frac{1+\epsilon }{\epsilon }\}$ for *ϵ* = 0.05, because GPs calculate latent values before predicting probabilities. Points where $\mu ({x}^{* }| {\mathcal{D}})\approx 0$ likely lie near the phase boundary; points with high predictive variance represent regions of high uncertainty. This approach requires spatial correlation in phase diagrams: points with similar parameter values tend to belong to the same phase. The covariance function $k(x,{x}^{{\prime} })$ encodes this assumption; we select the Matérn-3/2 kernel for its appropriate smoothness.

The active learning framework defines an acquisition function *a*(*x*) that quantifies the value of sampling at each candidate point. The standard acquisition function^20^ balances two objectives: exploiting current knowledge by sampling near the predicted phase boundary (*μ*(x^*^∣*D*) ≈ 0), and exploring uncertain regions where the model lacks confidence:

3 $$a({x}^{* }| D)=\frac{\sqrt{{\sigma }^{2}({x}^{* }| {\mathcal{D}})}}{| \mu ({x}^{* }| {\mathcal{D}})| +{\epsilon }_{acq}},$$

where we add *ϵ*_*acq*_ ≈ 0 for numerical stability.

At each iteration, the algorithm selects the next point *x*_*t*+1_ = *arg* *max* *a*(*x*), performs the corresponding experiment to obtain *y*_*t*+1_, and updates the GP posterior. This process continues until reaching a stopping criterion, such as sampling *n* points or achieving a KL divergence below a threshold for subsequent phase diagrams.

For notational convenience, we define the predictive probability output of a trained Gaussian process. Given a GP trained on dataset *D* and evaluated at point x*:

4 $$GP({x}^{* })=\Phi (\mu ({x}^{* }| {\mathcal{D}})),$$

where $\mu ({x}^{* }| {\mathcal{D}})$ is the posterior mean, and *Φ* is the standard normal CDF mapping the latent function value to a probability in [0, 1].

### PhaseTransfer transfer learning framework

Active learning with GPs efficiently maps phase diagrams but treats each system independently, ignoring valuable knowledge from previously studied related systems. Transfer learning (TL) addresses this limitation by utilizing information from source phase diagrams to accelerate characterization of new target systems. PhaseTransfer extends the above active learning framework by incorporating prior knowledge from one or more source phase diagrams, influencing both target system predictions and the acquisition function for selecting sampling points (Fig. 1a).

Single-Source PhaseTransfer combines three components to make predictions and acquisition value calculations:

1. A target model *GP*_*t*_: A GP trained on labeled points ${{\mathcal{D}}}_{t}$ from the target phase diagram, i.e., the phase diagram to be determined. While we assume this is a GP due to its superior performance in low data scenarios, any model capable of outputting phase probabilities and uncertainties is acceptable.
2. A source model *GP*_*s*_: A GP trained on labeled points ${{\mathcal{D}}}_{s}$ from a known related phase diagram. While we assume this is a GP for consistency, any model capable of outputting phase probabilities is acceptable.
3. A weight model *GP*_*w*_: A GP trained on labeled points ${{\mathcal{D}}}_{w}$ derived from target observations ${{\mathcal{D}}}_{t}$. Any model outputting phase probabilities is acceptable. This model predicts where the source model correctly identifies phases, expressed as a 0-to-1 probability. The labels in *D*_*w*_ indicate whether *GP*_*s*_ correctly predict each target datapoint: 1 for correct predictions, 0 otherwise:
  5 $${D}_{w}=\left\{(x,f(y)):(x,y)\in {{\mathcal{D}}}_{t}\right\}\,f(y)=\left\{\begin{array}{ll}1\, & \mathrm{round}(G{P}_{s}(x))=y\\ 0 & \mathrm{round}(G{P}_{s}(x))\,\ne\, y\,.\end{array}\right.$$

We use the source-weight model pair (*GP*_*s*_, *GP*_*w*_) in two ways: (i) constructing an informed prior for *GP*_*t*_, and (ii) predicting the target phase in combination with *GP*_*t*_. To construct the informed prior, we set the prior mean of *GP*_*t*_ to a weighted version of the source model’s posterior mean *μ*_*s*_(*x*∣*D*_*s*_), injecting source knowledge where *GP*_*s*_ prove reliable:

6 $$\begin{array}{l}G{P}_{w}(x)\to w\\ {\mu }_{t}(x)=w{\mu }_{s}(x| {{\mathcal{D}}}_{s})\,.\end{array}$$

Where the source model is reliable (*w* ≈ 1), the prior reflects source knowledge; in unreliable regions (*w* ≈ 0), the prior reverts to the standard uninformed case, *μ*_*t*_(*x*) = 0.

For the final prediction, we combine all three models through weighted addition:

7 $$\begin{array}{l}G{P}_{w}({x}^{* })\to w\\ {\rm{pred}}({x}^{* })={w}^{p(G{P}_{s}({x}^{* }))}G{P}_{s}({x}^{* })+(1-{w}^{p(G{P}_{s}({x}^{* }))})G{P}_{t}({x}^{* }),\end{array}$$

where *p*( ⋅ ) is an adaptive concave function mapping source predictions *GP*_*s*_(x^*^) to an exponent in [1, *m*] (with *m* = 5 controlling maximum exponent strength):

8 $$p(G{P}_{s}({x}^{* }))=\log (e+2({e}^{m}-e)| G{P}_{s}({x}^{* })-0.5| ).$$

This function links source confidence to exponent strength, down-weighting confident source predictions (*GP*_*s*_(x^*^) ≈ 0 or *GP*_*s*_(x^*^) ≈ 1) unless the source is trusted (*w* ≈ 1), thereby avoiding over-reliance on potentially misleading predictions. The specific functional form matters less than its qualitative behavior: amplifying the weight requirement as source confidence increases (SI Section 1, Fig. S1).

To optimize sampling strategy under TL, we modify the standard acquisition function (3):

9 $$a({x}^{* })={w}^{{\prime} }(\sqrt{{\sigma }_{t}^{2}({x}^{* }| {{\mathcal{D}}}_{t})}+d({x}^{* },{{\mathcal{D}}}_{t}))+(1-{w}^{{\prime} })\frac{\sqrt{{\sigma }_{t}^{2}({x}^{* }| {{\mathcal{D}}}_{t})}}{| {\mu }_{t}({x}^{* }| {{\mathcal{D}}}_{t})| +{\epsilon }_{acq}}$$

10 $${w}^{{\prime} }=\left\{\begin{array}{ll}2G{P}_{w}({x}^{* })-1 & \mathrm{if}G{P}_{w}({x}^{* }) > \tau =0.6\\ 0 & \mathrm{otherwise}\,,\end{array}\right.$$

where $d({x}^{* },{{\mathcal{D}}}_{t})$ represents the minimum Euclidean distance from x^*^ to existing target observations. This optional term discourages excessive sampling in small regions. The acquisition function balances two strategies: where source knowledge is highly trusted (*GP*_*w*_(x^*^) > *τ* = 0.6), it prioritizes exploring unsampled areas over redundantly characterizing known boundaries; where source confidence is lower, it defaults to the standard acquisition strategy (3). As with *p*( ⋅ ), the specific form of Eq. (10) matters less than its qualitative behavior: switching between exploration-focused and boundary-focused sampling based on source reliability (SI Section 1, Fig. S1).

Multi-Source PhaseTransfer extends Single-Source PhaseTransfer by maintaining a collection of source-weight model pairs. For each source model $G{P}_{s}^{i}$ trained on labeled source points ${D}_{s}^{i}$, we train a corresponding weight model $G{P}_{w}^{i}$ using Eq. (5), yielding *n* pairs: $\{(G{P}_{s}^{1},G{P}_{w}^{1}),\ldots ,(G{P}_{s}^{n},G{P}_{w}^{n})\}$.

For phase prediction at point x^*^, we select the most reliable source model (with index ${i}^{{\prime} }$):

11 $$\begin{array}{l}\arg \mathop{\max }\limits_{i}G{P}_{w}^{i}({x}^{* })\to {i}^{{\prime} }\\ G{P}_{w}^{{i}^{{\prime} }}({x}^{* })\to w\\ {\rm{pred}}({x}^{* })={w}^{p(G{P}_{s}^{{i}^{{\prime} }}({x}^{* }))}G{P}_{s}^{{i}^{{\prime} }}({x}^{* })+(1-{w}^{p(G{P}_{s}^{{i}^{{\prime} }}({x}^{* }))})G{P}_{t}({x}^{* }),\end{array}$$

where *p*( ⋅ ) is the adaptive exponent from Eq. (8). At each location, predictions rely on the source model with the highest predicted reliability, defaulting to the target model when all sources show low confidence.

We modify the informed prior to incorporate knowledge from all source models through a weighted sum:

12 $$\begin{array}{l}G{P}_{w}^{i}({x}^{* })\to {w}_{i}\\ {\mu }_{t}({x}^{* })=\mathop{\sum }\limits_{i}{w}_{i}^{p(G{P}_{s}^{i}({x}^{* }))}{\mu }_{s}^{i}({x}^{* }| {{\mathcal{D}}}_{s}^{i})\,.\end{array}$$

Here, *p*( ⋅ ) reduces overconfidence when multiple aligned source models contribute to the same region, ensuring the combined prior remains well-calibrated as source count increases.

The acquisition function follows Eq. (9) but uses the maximum weight across all source models for auxiliary weights ${w}^{{\prime} }$:

13 $$\begin{array}{l}\arg \mathop{\max }\limits_{i}G{P}_{w}^{i}(x)\to {i}^{{\prime} }\\ {w}^{{\prime} }=\left\{\begin{array}{ll}2G{P}_{w}^{{i}^{{\prime} }}({x}^{* })-1 & \mathrm{if}G{P}_{w}^{{i}^{{\prime} }}({x}^{* }) > \tau =0.6\\ 0 & \mathrm{otherwise}\,.\end{array}\right.\end{array}$$

This maintains the exploration-exploitation balance from the single source case while utilizing the best available source knowledge at each location.

### Source pruning

When working with multiple source phase diagrams, redundancy becomes a concern. Similar source models can produce overconfident predictions and increase computational cost, potentially degrading transfer learning performance. We implement an automated source pruning algorithm that selects a diverse subset of source models based on predicted phase diagram distinctiveness.

The pruning algorithm evaluates source models on a common grid using three complementary criteria:

1. Pixel-wise agreement: Measures overall agreement through accuracy between binary phase predictions. Two diagrams must exceed an accuracy threshold *τ*_*acc*_ = 0.9 to be considered potentially similar, ensuring conservative filtering.
2. Topological structure: Compares topological invariants using persistent homology, extracting the number of connected components and holes from each phase diagram. Two diagrams are considered potentially similar only if both invariants match exactly.
3. Boundary disagreement: Addresses small boundary variations arising from slight model differences. We compute a tolerant disagreement map using binary dilation:
  14 $$\begin{array}{l}{\rm{Agreement}}(A,B)=(A\cap {B}_{{\rm{dil}}})\cup (B\cap {A}_{{\rm{dil}}})\\ {\rm{Disagreement}}(A,B)=\neg {\rm{Agreement}}(A,B)\cap (A\cup B)\end{array}$$
  where *A*_dil_ and *B*_dil_ represent dilations of diagrams *A* and *B* with radius *r* = 5 units. The number of disconnected disagreement regions must remain below the threshold *t* = 3 for diagrams to be considered potentially similar.

Source selection follows a greedy algorithm: we evaluate source models sequentially, adding each to the selected ensemble only if it differs from all previously selected models by at least one criterion. This ensures the final ensemble contains diverse phase behaviors while eliminating redundant information.

### Benchmarks evaluation protocol

For each benchmark, we evaluate predictions on a test grid independent of the sampled points. Unless otherwise noted, this grid is a uniform 100 × 100 lattice spanning the parameter space.

All synthetic benchmarks follow a consistent protocol:

1. Define one target phase diagram and a set of source phase diagrams.
2. Generate source models by executing vanilla active learning on each source diagram, initializing with 5–10 randomly selected points and acquiring 50 additional samples.
3. Evaluate all methods on the target task using 5–10 initial points and 50–100 additional samples, recording misclassification error at each iteration.
4. Repeat across 50 independent runs with different random initializations to assess statistical significance. Within each run, all methods use identical random seeds, ensuring the same initial sampling points for fair comparison.

## Supplementary information

Supplementary Information

## Data Availability

The data supporting the findings of this study are available in the GitHub repository at https://github.com/BigChemistry-RobotLab/phaseGP.

## Code availability

The phaseGP Python library that integrates the described algorithms, as well as the scripts for reproducing our findings, is available in the following GitHub repository https://github.com/BigChemistry-RobotLab/phaseGP.
