# Supplementary material for: PhaseTransfer: A transfer learning framework for efficient phase diagram mapping
Source: NPJ Comput Mater. 2026 May 26;12(1):279. doi: 10.1038/s41524-026-02154-2 (PMC13485685; doi:10.1038/s41524-026-02154-2)
Supplement: Supplementary file 1 [file 41524_2026_2154_MOESM1_ESM.pdf]

Supplementary Information for: PhaseTransfer: A transfer learning framework for efficient phase diagram mapping

Eduardo González-García[1,2], Albert J. Markvoort[1,2], Nadia A. Erkamp[1,2], Tom F.A. de Greef[1,2,3,4,5]

[1]Synthetic Biology Group, Department of Biomedical Engineering, Eindhoven University of Technology, Eindhoven, The Netherlands
[2]Institute for Complex Molecular Systems, Department of Biomedical Engineering, Eindhoven University of Technology, Eindhoven, The Netherlands
[3]Laboratory of Chemical Biology, Department of Biomedical Engineering, Eindhoven University of Technology, Eindhoven, The Netherlands
[4]Institute for Molecules and Materials, Radboud University, Nijmegen, The Netherlands
[5]Center for Living Technologies, Eindhoven-Wageningen-Utrecht Alliance, Utrecht, The Netherlands

1 Hyperparameter Sensitivity Analysis

PhaseTransfer introduces two key hyperparameters: the maximum exponent strength m in the adaptive function $p(\cdot)$ (main text Equation 8) and the threshold τ in the acquisition function (main text Equation 13). To assess robustness to hyperparameter selection, we evaluate Multi-Source PhaseTransfer on the sine wave benchmark (main text Synthetic Benchmark: Sine Wave Phase Diagram) across a range of values for both m and τ.

For the adaptive exponent maximum strength m, we test values $m \in \{2, 3, 5, 7\}$ alongside a baseline without adaptive exponentiation (constant exponent of 1). Results across 50 independent runs show that all adaptive exponent variants achieve comparable performance, consistently outperforming the non-adaptive baseline (Figure S1a). This demonstrates that the qualitative behavior of amplifying weight requirements with source confidence matters more than the specific exponent strength.

For the exploration threshold τ in the acquisition function, we test values $\tau \in \{0.5, 0.6, 0.7, 0.8\}$ alongside the standard boundary-focused acquisition regardless of source reliability. All threshold values achieve similar mean performance, consistently outperforming the baseline without exploration enhancement, which also exhibits substantially higher variance (Figure S1b). This increased variability arises from the algorithm's reduced ability to discover novel features (specifically the circular boundary absent from source diagrams) when it fails to prioritize exploration in high-confidence regions.

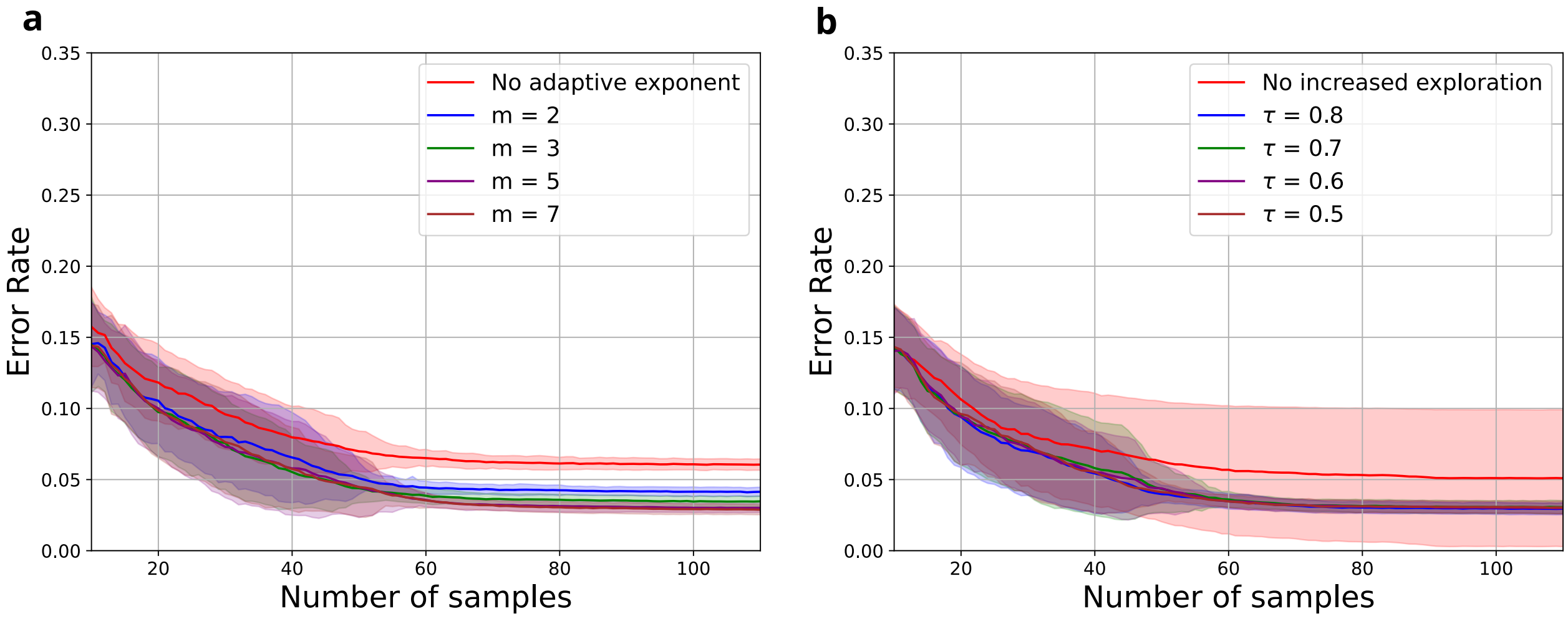

Figure S1: **Hyperparameter sensitivity analysis for the sine wave benchmark.** Error comparison of Multi-Source PhaseTransfer with varying hyperparameter values averaged over 50 independent runs. Error rate represents fraction of misclassified points on a 100×100 test grid. **(a)** Adaptive exponent maximum strength m: All adaptive variants ($m \in \{2, 3, 5, 7\}$) achieve comparable performance and consistently outperform the non-adaptive baseline (constant exponent), confirming robustness to the specific choice of m. **(b)** Exploration threshold τ: All threshold values ($\tau \in \{0.5, 0.6, 0.7, 0.8\}$) achieve similar mean convergence, but the baseline without exploration enhancement (standard boundary-focused acquisition only) shows substantially higher variance. The increased standard deviation reflects inconsistent discovery of the novel circular boundary, demonstrating that adaptive exploration improves reliability across different initial sampling configurations.

2 Three-Component Biological Condensate: Interaction Parameters

The Flory-Huggins interaction parameters χ_{PR}, χ_{PS}, and χ_{RS} govern the pairwise interactions between protein (P), RNA (R), and salt (S) in the three-component biological condensate benchmark (main text Synthetic Benchmark: Three-component Biological Condensate). These parameters depend on ionic strength I and, for χ_{PR}, on component concentrations:

$$\begin{cases} \chi_{PR}(I, \phi_P, \phi_R) = \left[\chi^0_{PR} + A\,(\phi_P + \phi_R)^2 - B\,(\phi_P + \phi_R)^4\right] e^I & ; \chi^0_{PR} = -1.8, A = 20, B = 30 \\ \chi_{PS}(I) = \chi^0_{PS} + k_{PS} I & ; \chi^0_{PS} = 0.1, k_{PS} = 0.3 \\ \chi_{RS}(I) = \chi^0_{RS} + k^0_{RS} I + k^1_{RS} I^2 & ; \chi^0_{RS} = -0.2, k^0_{RS} = 0.6, k^1_{RS} = -0.8\,. \end{cases} \tag{1}$$

These functional forms capture simplified representations of key physical interactions in biological condensates. The protein-RNA interaction χ_{PR} incorporates concentration-dependent effects that balance attractive molecular interactions against crowding-induced repulsion at high densities, with ionic strength modulation accounting for electrostatic screening. The protein-salt interaction χ_{PS} follows linear ionic strength dependence, representing standard salting-out behavior where increasing salt concentration weakens protein-solvent interactions. The RNA-salt interaction χ_{RS}

captures non-monotonic salting effects through quadratic ionic strength dependence. These specific mathematical forms matter less than their ability to generate phase diagrams with characteristic reentrant boundaries, enabling systematic testing of transfer learning across related systems.

3 Supramolecular Copolymerization: Mass-balance Equations

The supramolecular copolymerization benchmark (main text Synthetic Benchmark: Supramolecular Copolymerization) considers a two-monomer system (A and B) undergoing nucleation–elongation polymerization where B–B bond formation is forbidden. Supramolecular polymerization proceeds through reversible association reactions between monomers and aggregates. The key equilibrium steps are:

Nucleation (dimer formation):

$$\begin{aligned} A + A &\rightleftharpoons AA \quad K = \sigma K_{AA}, \\ A + B &\rightleftharpoons AB \quad K = \sigma K_{AB}, \\ B + A &\rightleftharpoons BA \quad K = \sigma K_{BA}. \end{aligned} \tag{2}$$

Elongation (chain growth):

$$\begin{aligned} P_zA + A &\rightleftharpoons P_zAA \quad K = K_{AA}, \\ P_zA + B &\rightleftharpoons P_zAB \quad K = K_{AB}, \\ P_zB + A &\rightleftharpoons P_zBA \quad K = K_{BA}. \end{aligned} \tag{3}$$

Here P_z represents an arbitrary copolymer of length $z \geq 1$, K_{XY} are elongation equilibrium constants for bonds between monomers $X, Y \in \{A, B\}$, and $\sigma \ll 1$ introduces the nucleation penalty. We employ the mass-balance equations derived by Eikelder et al. [45] to compute equilibrium polymer concentrations from these reactions using parameters specified in main text Equation (2).

In this framework, each monomer type exists either as free monomers (a or b) or incorporated within aggregates. The equivalent concentration of a monomer type within polymers represents the total concentration of that monomer bound in all aggregates, expressed as if those monomers were free. Thus, $P_A(a, b)$ and $P_B(a, b)$ denote the equivalent concentrations of monomers A and B in polymers of all lengths, respectively. Given initial monomer concentrations a_{tot} and b_{tot}, the total polymer concentration $P_{tot} = P_A + P_B$ follows from solving:

$$\begin{cases} a + P_A(a, b) = a_{tot} \\ b + P_B(a, b) = b_{tot} \ . \end{cases} \tag{4}$$

Computing $P_A(a, b)$ and $P_B(a, b)$ requires accounting for monomers in aggregates of all sizes. We represent concentration information through four-component vectors $\mathbf{u}_n^X$ encoding information for polymers of length n. The first two components represent concentrations of chains ending with A or B respectively, while the third and fourth components represent the equivalent concentrations of monomer $X \in \{A, B\}$ within chains ending with A or B respectively. $\mathbf{u}_n^X$ is obtained through a

set of recursive operations starting at a seed vector $\mathbf{u}_1^X$:

$$\mathbf{u}_1^X = \begin{cases} (\sigma a, \sigma b, \sigma a, 0) & \text{if } X = A \\ (\sigma a, \sigma b, 0, \sigma b) & \text{if } X = B\,. \end{cases} \tag{5}$$

We encode the polymerization mechanism through matrices constructed from equilibrium constants in main text Equation (2):

$$M_A = \begin{pmatrix} K_{AA}a & K_{BA}a \\ 0 & 0 \end{pmatrix} = K_e \begin{pmatrix} f_{AA}a & f_{AB}a \\ 0 & 0 \end{pmatrix} \tag{6}$$

$$M_B = \begin{pmatrix} 0 & 0 \\ K_{AB}b & K_{BB}b \end{pmatrix} = K_e \begin{pmatrix} 0 & 0 \\ f_{AB}b & 0 \end{pmatrix} \tag{7}$$

$$M_c = \begin{pmatrix} K_{AA}a & K_{BA}a \\ K_{AB}b & K_{BB}b \end{pmatrix} = K_e \begin{pmatrix} f_{AA}a & f_{AB}a \\ f_{AB}b & 0 \end{pmatrix}\,. \tag{8}$$

Then, $\mathbf{u}_n^X$ is calculated by recursively multiplying these matrices:

$$\mathbf{u}_n^X = M_{T,X}^{n-1}\,\mathbf{u}_1^X \quad \text{with} \quad M_{T,X} = \begin{pmatrix} M_c & 0 \\ M_X & M_c \end{pmatrix}, \quad X \in \{A, B\}\,, \tag{9}$$

where each matrix application computes polymer concentrations and equivalent concentrations for chains of length n from those of length $n-1$. The total equivalent concentration of X in polymers ending with A and those ending with B (P_X) sums contributions from all aggregate sizes, extracting the third and fourth vector components:

$$P_X(a,b) = \sum_{n=2}^{\infty} \left(u_{n,3}^X + u_{n,4}^X\right) = U_3^X + U_4^X \tag{10}$$

$$\mathbf{U}^X = \sum_{n=2}^{\infty} \mathbf{u}_n^X = \sum_{n=1}^{\infty} M_{T,X}^n\,\mathbf{u}_1^X = (I - M_{T,X})^{-1}\, M_{T,X}\,\mathbf{u}_1^X\,. \tag{11}$$

The geometric series converges because under equilibrium conditions all eigenvalues of $M_{T,X}$ have absolute value less than 1. This enables efficient computation without explicit summation. We solve the nonlinear system for free monomer concentrations (a, b) using two nested bisection methods in Python, then compute P_{tot} from the polymer concentration functions. The binary phase assignment (main text Synthetic Benchmark: Supramolecular Copolymerization) classifies compositions where P_{tot} exceeds half the total monomer concentration $(a_{tot} + b_{tot})/2$ as the polymerized phase.

4 Extended Performance Evaluation

4.1 Comparison Against Space-Filling Sampling

Active learning derives its efficiency from acquisition functions that exploit model uncertainty estimates to direct sampling toward informative regions of parameter space. An alternative class of methods, space-filling designs, selects points based solely on geometric coverage of the parameter space, without reference to any model. To assess the benefit of uncertainty-guided sampling over geometry-based approaches, we compare our active learning method (uncertainty-guided) against

three space-filling methods, each tested with two models: vanilla GP and Multi-Source PhaseTransfer. This modular design isolates the contributions of the sampling strategy and the transferred prior. The three space-filling strategies are: (1) random sampling, which draws points uniformly at random from the parameter space and serves as an uninformed baseline; (2) Latin hypercube sampling (LHS)[28], which partitions each parameter dimension into equal-probability intervals and places exactly one sample per interval combination, ensuring more uniform marginal coverage than pure random sampling; and (3) minimax distance sampling [29], which selects points such that the maximum distance from any unsampled location to its nearest sampled point is minimized, providing a geometric coverage guarantee by filling the largest gaps in the parameter space. For each combination of model and sampling strategy, we use the same total sample budget and initialize from identical random starting points across 50 independent runs on the sine wave benchmark (main text Synthetic Benchmark: Sine Wave Phase Diagram). Figure S2a shows all the sampling strategies under the vanilla GP model; Figure S2b shows the same strategies under the Multi-Source PhaseTransfer model. This allows direct comparison of (1) active learning versus space-filling within each model, and (2) the effect of the transferred prior on each sampling strategy.

Results demonstrate that uncertainty-guided acquisition consistently outperforms space-filling strategies under both models (Figure S2). For the vanilla model (Figure S2a), active learning initially exhibits comparable performance to space-filling methods, as the model has yet to develop reliable uncertainty estimates. Once enough observations are accumulated and the phase boundary structure becomes better resolved, the acquisition function successfully concentrates sampling on informative regions, after which vanilla active learning diverges from and outperforms all three space-filling strategies. Notably, space-filling methods paired with source knowledge (Figure S2b) substantially outperform vanilla active learning (red) in early iterations, demonstrating that transferred information alone provide significant benefit regardless of sampling strategy. However, Multi-Source PhaseTransfer (green) still outperforms these space-filling methods, confirming that uncertainty-guided acquisition provides additional benefit beyond the transferred knowledge. Together, these results demonstrate that the performance gains of PhaseTransfer stem from the combination of informative priors and uncertainty-guided acquisition, and that neither component alone achieves optimal sampling efficiency.

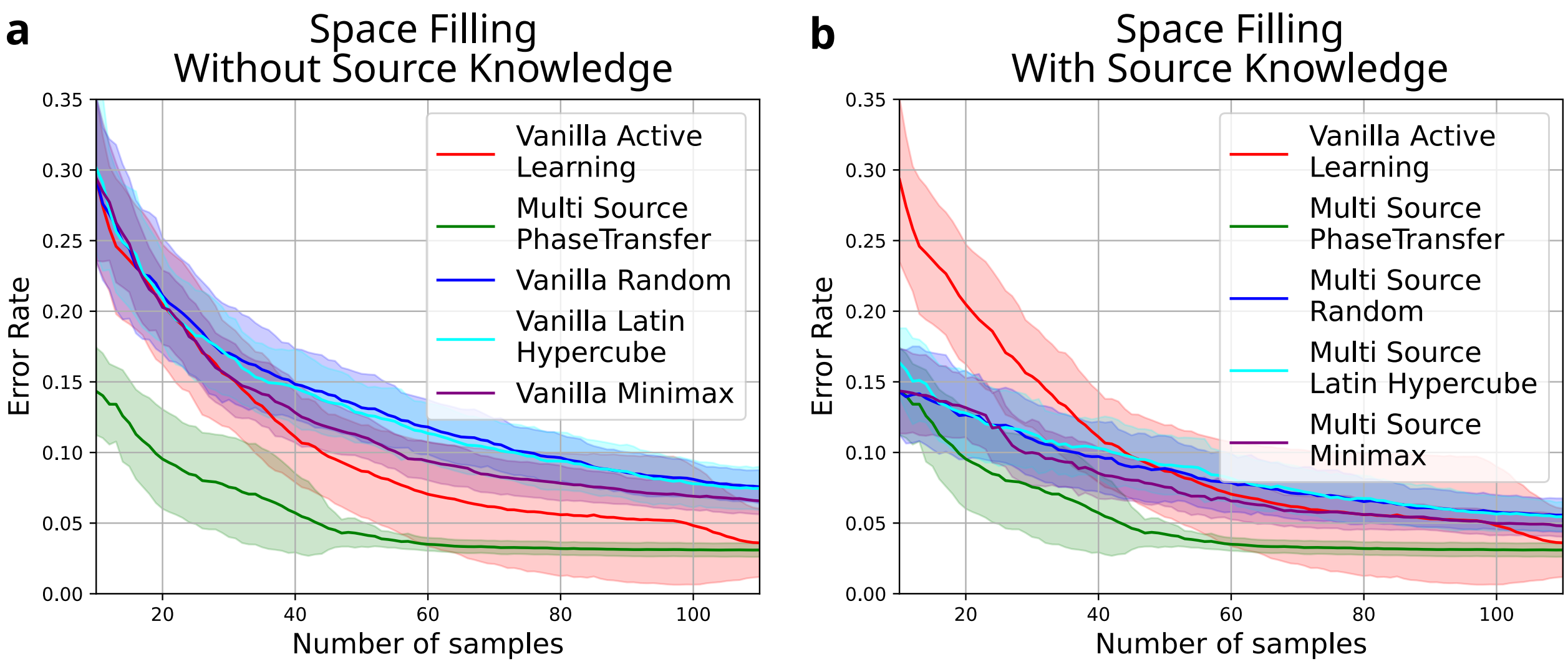

Figure S2: **Comparison of uncertainty-guided acquisition against space-filling sampling strategies on the sine wave benchmark.** Error comparison averaged over 50 independent runs with random initialization. Shaded regions show ± 1 standard deviation. Error rate represents fraction of misclassified points on a 100×100 test grid. **(a)** Vanilla active learning (red) and Multi-Source PhaseTransfer (green) versus random sampling, Latin hypercube sampling, and minimax distance sampling without source knowledge. Vanilla active learning initially performs comparably to space-filling methods but outperforms all three once sufficient observations allow the acquisition function to focus on informative regions. **(b)** Multi-Source PhaseTransfer (green) and vanilla active learning (red) versus the same three space-filling strategies with source knowledge. Space-filling methods with source knowledge substantially outperform vanilla active learning in early iterations, demonstrating the benefit of transferred information alone. However, Multi-Source PhaseTransfer still outperforms these space-filling methods, confirming that uncertainty-guided acquisition provides additional benefit beyond the prior knowledge.

4.2 Comparison Against Multi-Task Gaussian Process

Multi-task Gaussian processes (MultiTaskGPs)[30] represent an established approach to transfer learning in regression-based Bayesian optimization [31], where tasks are jointly modelled within a single GP. To assess whether PhaseTransfer offers advantages over this alternative in a phase mapping context, we implement a MultiTaskGP using our vanilla GP model (without Transfer Learning) as the base and evaluate it against PhaseTransfer on the sine wave benchmark (main text Synthetic Benchmark: Sine Wave Phase Diagram). The MultiTaskGP employs an Intrinsic Coregionalization Model (ICM) kernel, constructed as the Hadamard product of a standard Matérn-3/2 feature kernel and a learned task covariance kernel (IndexKernel), which encodes inter-task correlations. The model uses a variational approximation with inducing points drawn from the combined source and target observations, where target points receive task index 0 and source points receive indices $1, \ldots, K$. At inference time, query points are evaluated under task index 0, recovering target task predictions with the same interface as the vanilla GP model, making the MultiTaskGP directly compatible with the active learning acquisition loop. We evaluate both single-source and Multi-Source configurations against Single- and Multi-Source PhaseTransfer and vanilla active learning initialized from identical random starting points across 50 independent runs.

Results demonstrate that MultiTaskGP consistently underperforms PhaseTransfer across both single- and Multi-Source configurations (Figure S3). The error curves of MultiTaskGP exhibit higher instability than those of PhaseTransfer. A key limitation is the inability of MultiTaskGP to focus sampling on regions that cannot be explained by source knowledge: unlike PhaseTransfer, which shows a characteristic variance and error reduction associated with the active discovery of the novel circular boundary absent from all source diagrams, MultiTaskGP fails to exhibit this behavior. Furthermore, Multi-Source MultiTaskGP provides negligible improvement over its single-source variant, in contrast to the clear benefit observed when incorporating additional sources in PhaseTransfer. This indicates that the coregionalization framework struggles to effectively separate and exploit complementary information from multiple source tasks, instead diluting their contributions within the shared latent structure. Overall, MultiTaskGP performance converges to that of vanilla active learning, demonstrating that joint multi-task training does not translate into meaningful sampling efficiency gains for binary phase diagram mapping when compared to the spatially adaptive source weighting employed by PhaseTransfer.

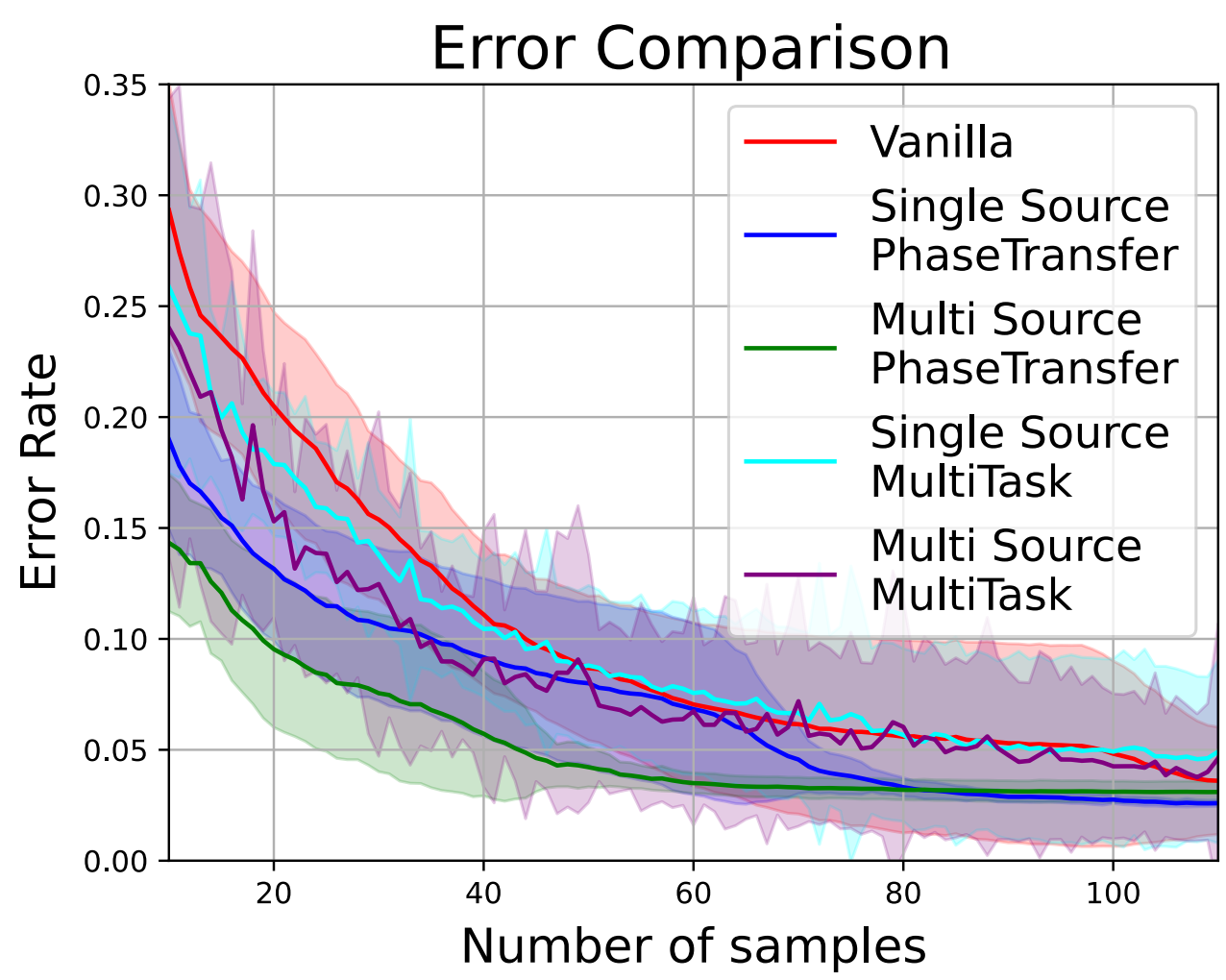

Figure S3: **Comparison of MultiTaskGP and PhaseTransfer on the sine wave benchmark.** Error comparison of vanilla active learning (red), Single- (dark blue) and Multi-Source PhaseTransfer (green), and Single- (light blue) and Multi-Source MultiTaskGP (purple) averaged over 50 independent runs with random initialization. Shaded regions show ±1 standard deviation. Error rate represents fraction of misclassified points on a 100×100 test grid. MultiTaskGP variants exhibit higher instability than PhaseTransfer, show negligible improvement from single- to Multi-Source configurations, and converge to performance comparable to vanilla active learning.

4.3 Robustness to Noise

To assess the robustness of PhaseTransfer against experimental noise, we evaluate the sinusoidal benchmark (main text Synthetic Benchmark: Sine Wave Phase Diagram) under noisy observations by adding measurement noise to both source and target tasks. The noise model includes two components that are applied independently to each sampled point:

- **Phase Noise:** Each sampled point has a low (2.5%) or high (5%) probability of misclassification, where the measured phase flips to the opposite class.

- **Input Shift:** Before phase assignment, each sampled point's coordinates shift randomly within a low (±5%) or high (±10%) interval of the total variable range.

Results across six noise combinations, evaluated over 50 independent runs from identical random starting points, demonstrate that all methods degrade under any noise regime, with input shift contributing more to performance degradation than phase misclassification noise, reflecting the greater disruption caused by spatial uncertainty in boundary localization relative to occasional label errors. Nevertheless, Multi-Source PhaseTransfer consistently outperforms baseline approaches across all six configurations (Figure S4).

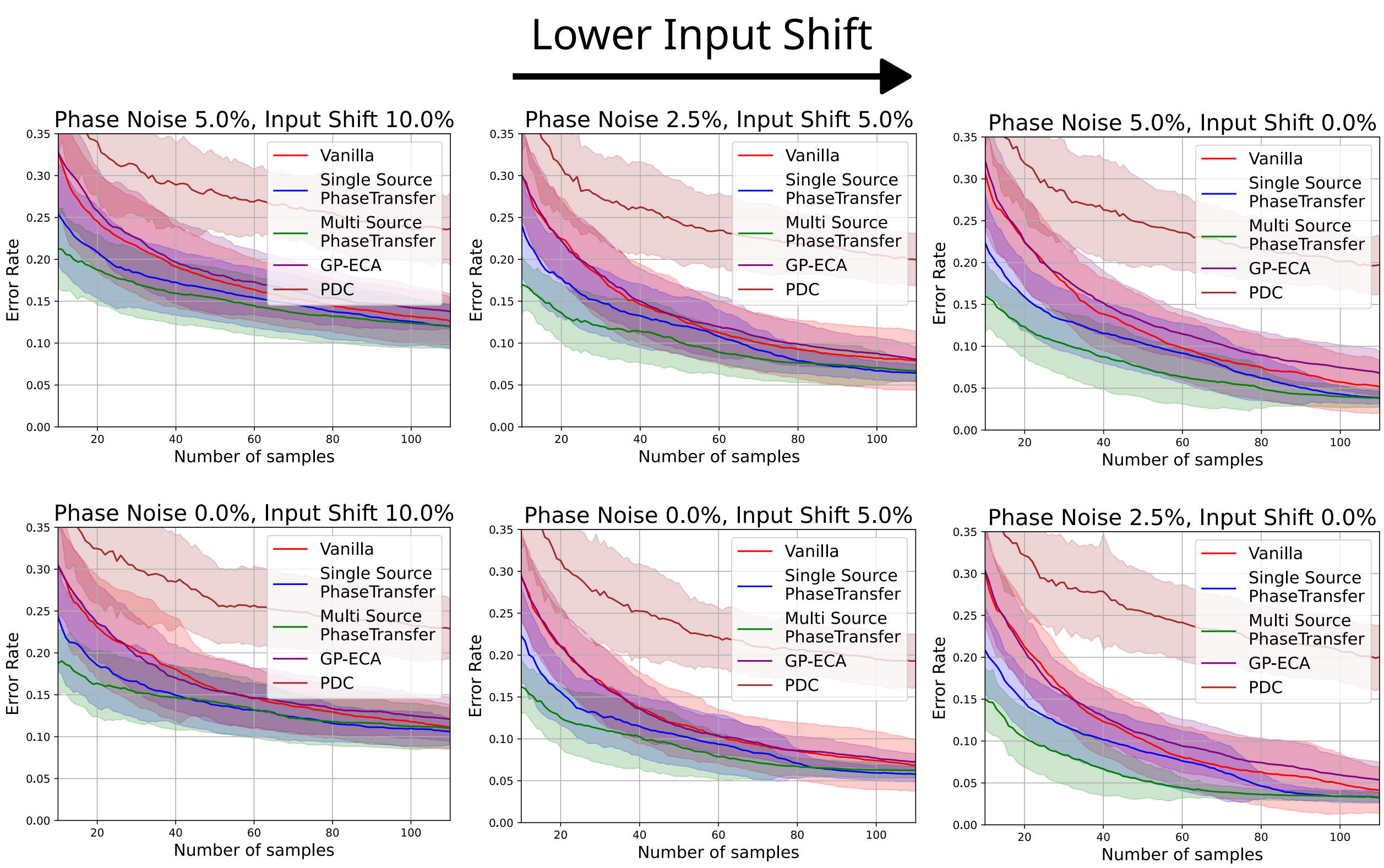

Figure S4: **Noisy sine wave benchmark results.** Error comparison across six noise configurations and five active learning methods, averaged over 50 independent runs with random initialization. Shaded regions show ±1 standard deviation. Error rate measured as fraction of misclassified points on a 100×100 grid test set. PhaseTransfer methods exhibit lower initial error and converge to comparable final accuracy. Multi-Source PhaseTransfer (green) consistently outperforms baseline methods throughout the active learning.

4.4 Source Variation Analysis

To assess how transfer learning performance varies with source-target similarity, we evaluate Single-Source PhaseTransfer under two controlled tests in which source-target boundary alignment is systematically degraded. Both tests isolate the effect of source quality by using a single source task, allowing direct attribution of performance changes to source-target mismatch.

In the first test, we use the sine wave benchmark (main text Synthetic Benchmark: Sine Wave Phase Diagram) with sources that share the same period as the target but lack the circular boundary. A

shift parameter controls the phase offset between source and target sinusoidal boundaries, ranging from 0 (no shift offset) to 1 (maximum shift of π radians), with intermediate values of 0.25, 0.5, and 0.75 (Figure S5a). This test isolates the effect of shifted source boundaries on transfer learning performance. In the second test, the target phase diagram consists of a single diagonal boundary with no circular feature. Source phase diagrams also consist of diagonal boundaries, but at a specified fraction of the boundary length the decision boundary deviates at a 90-degree angle, introducing a controlled fraction of misalignment between source and target. The fraction of the boundary that deviates is varied across $\{0, 0.25, 0.5, 0.75, 1\}$, where 0 corresponds to a perfectly aligned source and 1 corresponds to complete misalignment (Figure S5c). Together, these two tests provide complementary perspectives on source degradation: the first captures globally shifted but otherwise similar boundaries, while the second captures locally exact but partially misaligned boundaries.

Results from identical random starting points across 50 independent runs demonstrate that source degradation progressively reduces Single-Source PhaseTransfer performance in both tests, with the rate of degradation depending on the geometric complexity of the misaligned regions. In the sine wave shift test (Figure S5b), perfect source-target alignment yields the strongest performance, as the framework only needs to discover the novel circular boundary absent from the source. Once a phase offset is introduced, performance degrades rapidly beyond a shift of 0.25, because the complex intersections between shifted sinusoidal source and target boundaries make it difficult for the weight models to accurately characterize the unreliable regions. Notably, all non-zero offsets require a comparable number of iterations to resolve these complex unreliable regions to reach an error rate below 0.05, but they all outperform the vanilla baseline in both efficiency and variance. In the diagonal misalignment test (Figure S5d), performance degrades linearly with misalignment fraction, requiring between 4 and 6 additional iterations per 25% increase in misalignment, until complete misalignment returns performance equivalent to vanilla active learning. The absence of complex source-target boundary intersections in this test makes the unreliable regions easier to learn, explaining why degradation follows a linear rather than rapid pattern. Together, these results indicate that Single-Source PhaseTransfer efficiency depends jointly on the degree of source-target boundary alignment and on the geometric complexity of the regions where source knowledge proves unreliable. This degrading behavior is expected to be mitigated when multiple sources are incorporated into the ensemble (main text Synthetic Benchmark: Sine Wave Phase Diagram), as the framework then distributes reliance across sources rather than depending on accurate characterization of the unreliable regions of a single source.

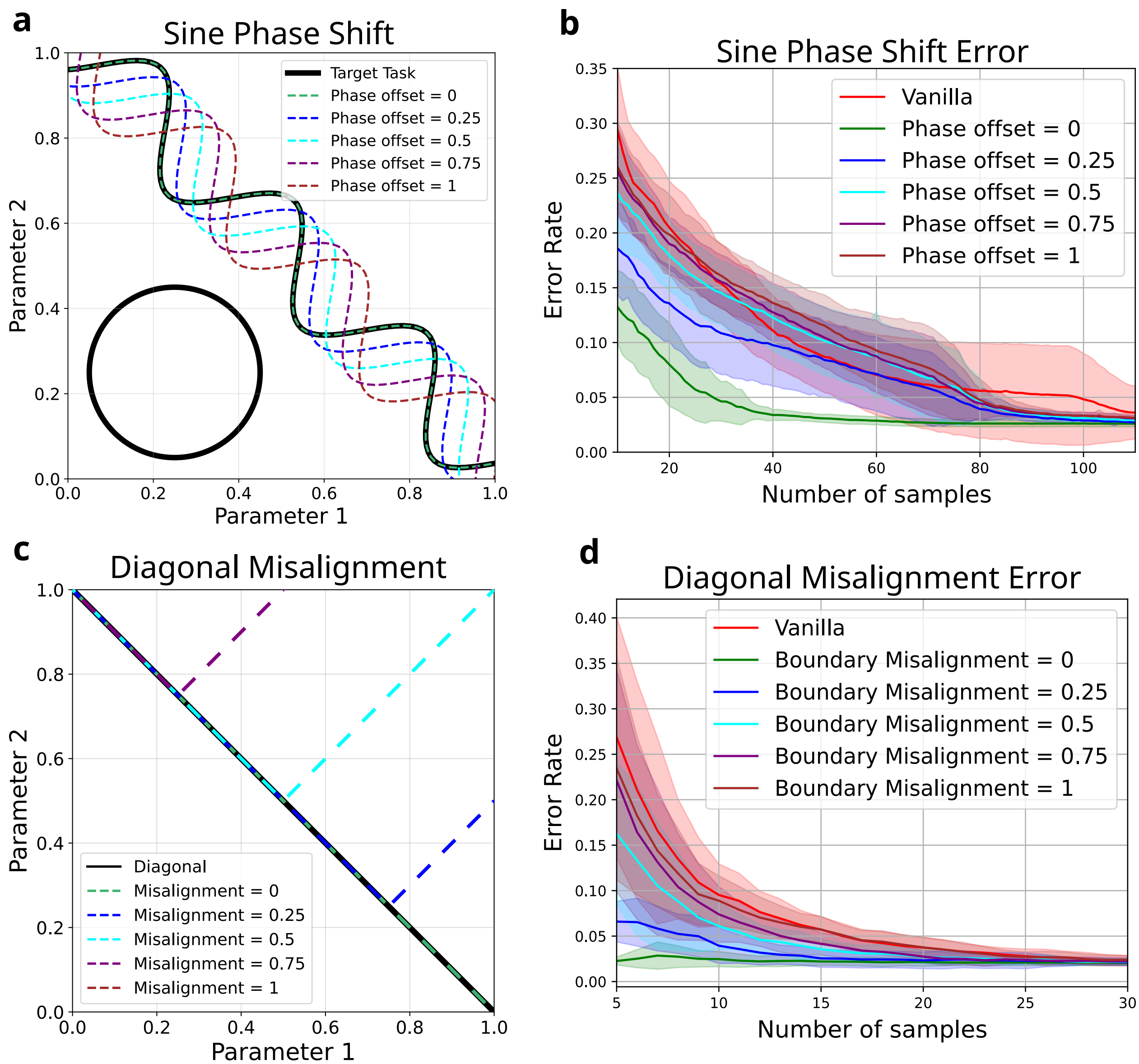

Figure S5: **Source variation analysis for Single-Source PhaseTransfer under controlled source-target misalignment.** **(a)** Schematic of sine phase boundaries of target (solid) and five sources (dashed) with phase shift offsets ranging from perfect alignment (0) to maximum misalignment (1). **(b)** Error comparison of Single-Source PhaseTransfer with phase offsets $\in \{0, 0.25, 0.5, 0.75, 1\}$ against vanilla active learning, averaged over 50 independent runs with random initialization. Shaded regions show ± 1 standard deviation. Error rate represents fraction of misclassified points on a 100×100 test grid. Performance degrades rapidly beyond an offset of 0.25 due to complex source-target boundary intersections, yet all non-zero offsets remain consistently faster than vanilla once unreliable regions are resolved. **(c)** Schematic of diagonal phase boundaries of target (solid) and five sources (dashed) with misalignment fractions ranging from perfect alignment (0) to maximum misalignment (1). **(d)** Error curves for the diagonal misalignment test with misalignment fractions $\in \{0, 0.25, 0.5, 0.75, 1\}$ against vanilla active learning. Performance degrades linearly, requiring between 4 to 6 additional iterations per 25% increase in misalignment, until complete misalignment returns performance equivalent to vanilla active learning.

4.5 Robustness to Irrelevant Sources

Transfer learning risks negative transfer when source tasks provide irrelevant information. To assess whether PhaseTransfer can identify and downweight such sources, we use the supramolecular copolymerization benchmark (main text Synthetic Benchmark: Supramolecular Copolymerization) with varying numbers of relevant and irrelevant source tasks. We test Multi-Source PhaseTransfer with n relevant sources (main text Synthetic Benchmark: Supramolecular Copolymerization, Matthews correlation coefficients of 0.82 and 0.77 with the target) and m irrelevant sources, where $n \in \{0, 1, 2\}$ and $m \in \{0, 1, 2, 4\}$. For $m \in \{1, 2\}$, irrelevant sources correspond to shifted diagonal boundaries (Matthews correlation coefficients of -0.37 and -0.43 with the target). For $m = 4$, two additional irrelevant sources are included that constantly predict phase 0 and phase 1 respectively (Matthews correlation coefficient of 0 by definition), representing a more adversarial regime in which uncorrelated sources also output highly confident predictions (Figure S6a). Note that $n = 0, m = 0$ corresponds to vanilla active learning without transfer learning.

Results from identical random starting points across 50 independent runs reveal that for $m \in \{0, 1, 2\}$, error curves cluster into three distinct groups based solely on the number of correlated sources, regardless of uncorrelated source count (Figure S6b). However, increasing to $m = 4$ with the additional constant prediction models produces a clear performance degradation across all values of n, partially attributable to the highly confident but incorrect predictions output by the constant source models. The degree of robustness under this adversarial regime depends strongly on the number of relevant sources available. With $n = 2$, performance initially degrades but recovers by approximately iteration 22, after which the framework successfully nullifies the influence of the uncorrelated sources and converges with the lower-m curves, showing no considerable impact on the final mapping efficiency. With $n = 1$, the initial performance remains above the vanilla baseline, but the framework becomes increasingly saturated by uncorrelated information throughout the active learning process, resulting in comparable efficiency to the vanilla baseline (with no irrelevant sources). With $n = 0$, the absence of any relevant source leaves the framework fully exposed to uncorrelated information, producing the strongest performance degradation and clearly underperforming the vanilla baseline. These results confirm that incorporating multiple relevant sources is the most effective mitigation against this failure mode, and that under realistic conditions, where at least a small number of genuinely related source diagrams are available, PhaseTransfer maintains its advantage over vanilla active learning even in the presence of adversarial source knowledge.

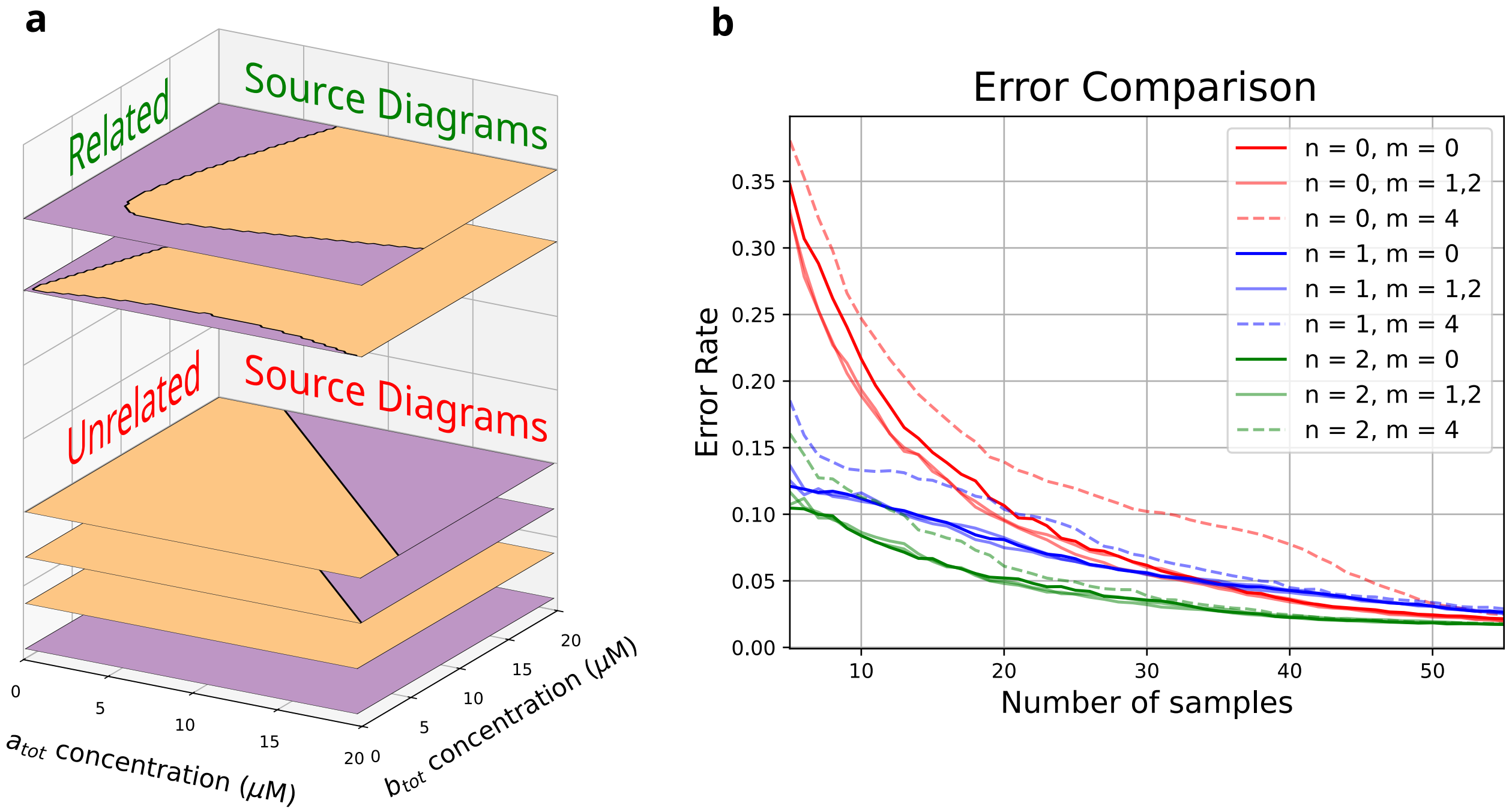

Figure S6: **Robustness to irrelevant sources in the supramolecular copolymerization benchmark.** **(a)** Source diagram library showing related and unrelated phase diagrams. Top: Two related source diagrams exhibiting strong correlation with the target system (Matthews correlation coefficients of 0.82 and 0.77). Bottom: Four unrelated source diagrams with top two being shifted diagonal boundaries showing negative correlation with the target (Matthews correlation coefficients of -0.37 and -0.43) and bottom two being constant prediction sources predicting phase 0 and phase 1 respectively (Matthews correlation coefficient of 0). **(b)** Error comparison for Multi-Source PhaseTransfer configurations varying the number of correlated ($n \in \{0, 1, 2\}$) and uncorrelated ($m \in \{0, 1, 2, 4\}$) source tasks. Results averaged over 50 independent runs from identical random starting points. Error rate represents fraction of misclassified points on a 100×100 test grid. For clarity, curves with $m = 1$ and $m = 2$ use the same shaded color band per n value. For $m \in \{0, 1, 2\}$, error curves cluster into three groups based solely on n, independent of m. For $m = 4$, performance degrades across all n values, with robustness increasing with the number of relevant sources.

4.6 Batch Sampling Performance

Experimental platforms often sample in batches to improve throughput. To verify that our active learning methods maintain performance under batch acquisition, we evaluate all three synthetic benchmarks using both individual sampling (one point per iteration) and batch sampling (10 points per iteration). For batch sampling, we select points that maximize the acquisition function while maintaining minimum separation distance $r = 0.1$ in normalized coordinates.

Results across 50 independent runs demonstrate that batch sampling achieves comparable performance to individual sampling with modest increases in required sample counts (Figure S7). However, batch acquisition offers substantial practical advantages for high-throughput platforms where parallel measurements dramatically reduce total experimental time, confirming that PhaseTransfer remains effective when adapted for automated experimental workflows.

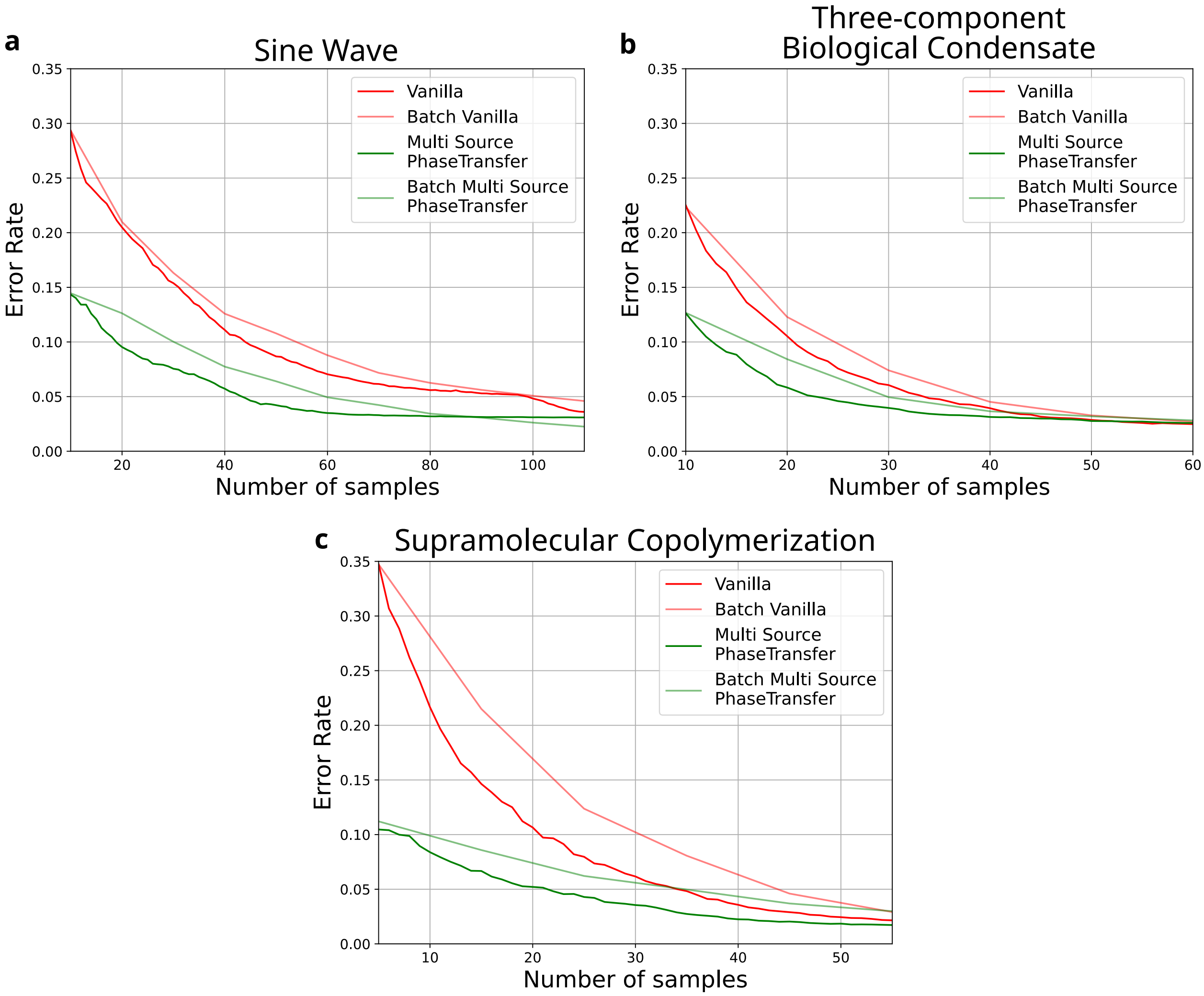

Figure S7: **Comparison of individual versus batch sampling across synthetic benchmarks.** Error comparison of vanilla active learning and Multi-Source PhaseTransfer under individual sampling (one point per iteration) and batch sampling (10 points per iteration) for **(a)** sine wave phase diagram, **(b)** three-component biological condensate, and **(c)** supramolecular copolymerization benchmarks. Results averaged over 50 independent runs. Error rate represents fraction of misclassified points on a 100×100 test grid in (a) and (c), and a cropped 140x140 triangular grid in in (b). Multi-Source PhaseTransfer consistently outperforms vanilla active learning in both sampling modes. Batch sampling exhibits modest sample efficiency degradation compared to individual sampling but offers significant advantages for high-throughput experimental platforms where parallel measurements reduce total experiment time.

5 phaseXplorer Observed Phase Separation

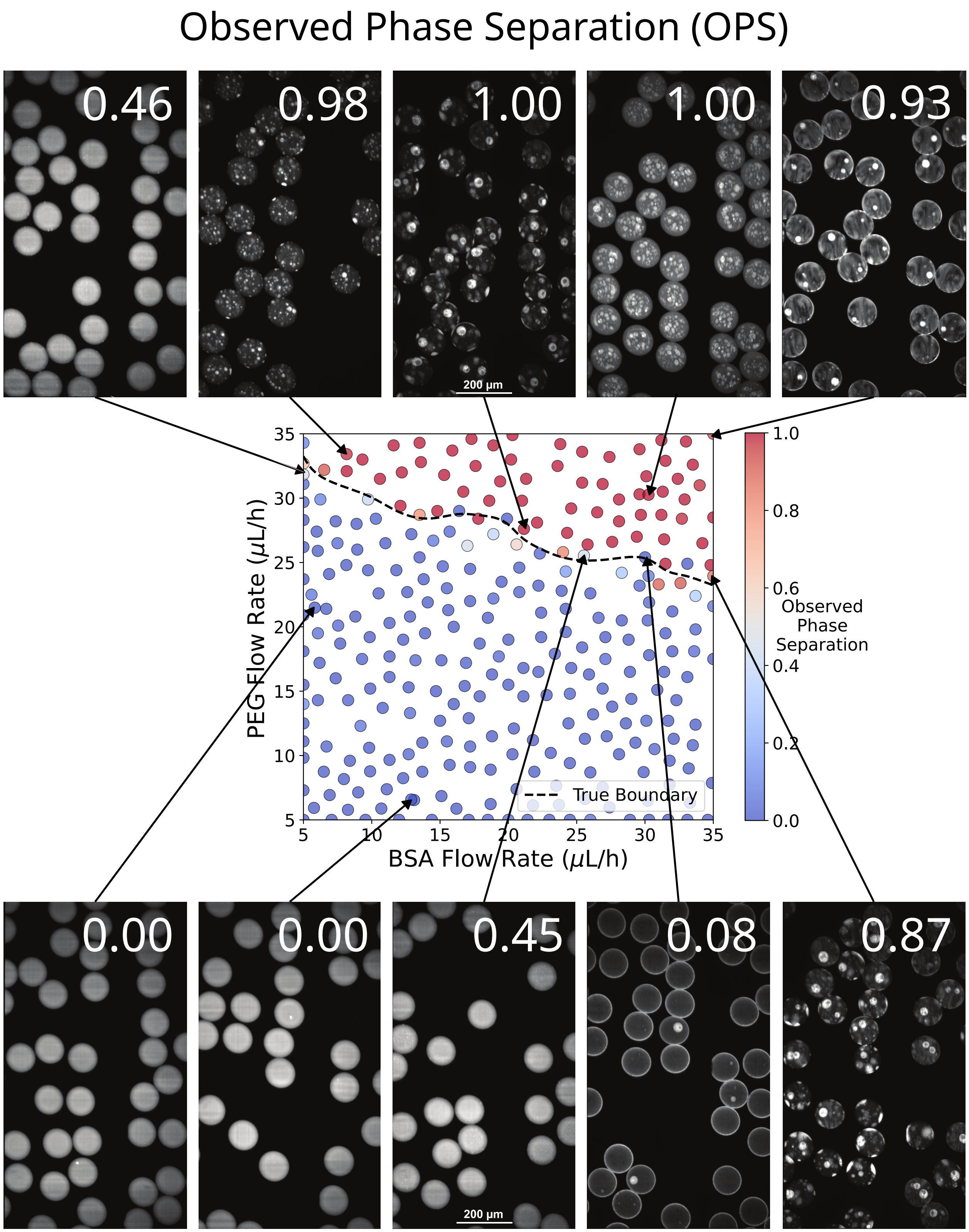

Figure S8: **Example PhaseXplorer confocal images with observed phase separation fraction.** Representative confocal microscopy images of microfluidic droplets at ten different compositions (top and bottom rows) with their corresponding observed phase separation fractions (top right corners). Arrows connect each image to its location on the ground truth phase diagram (center), established through exhaustive grid sampling of BSA-PEG mixtures at PEG 8k/pH 4.7/0 mM KCl. Color intensity on the diagram represents autonomously measured phase separation (blue: homogeneous, red: phase-separated). The images demonstrate PhaseXplorer's ability to accurately detect phase separation across the composition space: droplets in the phase-separated region (upper right, red) show distinct bright and dim areas corresponding to BSA-enriched condensates, while droplets in the homogeneous region (lower left, blue) exhibit uniform fluorescence. Quantitative assessment of measurement accuracy is provided in the main manuscript.
